# Small extrachromosomal circular DNAs as biomarkers for multi‐cancer diagnosis and monitoring

**DOI:** 10.1002/ctm2.1393

**Published:** 2023-08-30

**Authors:** Xuanmei Luo, Lili Zhang, Jian Cui, Qi An, Hexin Li, Zaifeng Zhang, Gaoyuan Sun, Wei Huang, Yifei Li, Chang Li, Wenzhuo Jia, Lihui Zou, Gang Zhao, Fei Xiao

**Affiliations:** ^1^ Peking University Fifth School of Clinical Medicine Beijing Hospital National Center of Gerontology Beijing China; ^2^ The Key Laboratory of Geriatrics Beijing Institute of Geriatrics Institute of Geriatric Medicine Chinese Academy of Medical Sciences Beijing Hospital National Center of Gerontology of National Health Commission Beijing China; ^3^ Clinical Biobank Beijing Hospital National Center of Gerontology National Health Commission Institute of Geriatric Medicine Chinese Academy of Medical Sciences Beijing China; ^4^ Department of General Surgery Beijing Hospital Beijing China; ^5^ National Center of Gerontology Institute of Geriatric Medicine Chinese Academy of Medical Sciences Beijing China

**Keywords:** biomarker, multi‐cancer analysis, nanopore sequencing, small extrachromosomal circular DNA

## Abstract

**Background:**

Small extrachromosomal circular DNAs (eccDNAs) have the potential to be cancer biomarkers. However, the formation mechanisms and functions of small eccDNAs selected in carcinogenesis are not clear, and whether the small eccDNA profile in the plasma of cancer patients represents that in cancer tissues remains to be elucidated.

**Methods:**

A novel sequencing workflow based on the nanopore sequencing platform was used to sequence naturally existing full‐length small eccDNAs in tissues and plasma collected from 25 cancer patients (including prostate cancer, hepatocellular carcinoma and colorectal cancer), and from an independent validation cohort (including 7 cancer plasma and 14 healthy plasma).

**Results:**

Compared with those in non‐cancer tissues, small eccDNAs detected in cancer tissues had a significantly larger number and size (*P* = 0.040 and 2.2e‐16, respectively), along with more even distribution and different formation mechanisms. Although small eccDNAs had different general characteristics and genomic annotation between cancer tissues and the paired plasma, they had similar formation mechanisms and cancer‐related functions. Small eccDNAs originated from some specific genes had great multi‐cancer diagnostic value in tissues (AUC ≥ 0.8) and plasma (AUC > 0.9), especially increasing the accuracy of multi‐cancer prediction of CEA/CA19‐9 levels. The high multi‐cancer diagnostic value of small eccDNAs originated from *ALK*&*ETV6* could be extrapolated from tissues (AUC = 0.804) to plasma and showed high positive predictive value (100%) and negative predictive value (82.35%) in a validation cohort.

**Conclusions:**

As independent and stable circular DNA molecules, small eccDNAs in both tissues and plasma can be used as ideal biomarkers for cost‐effective multi‐cancer diagnosis and monitoring.

## Graphical abstract

1. An efficient sequencing platform for full‐length small eccDNAs has been constructed.

2. Less than 0.01% small eccDNAs are shared in plasma and cancer tissue of the same patient.

3. Small eccDNAs show high multi‐cancer diagnostic value in tissues and plasma, especially when combined with CEA/CA19‐9 levels

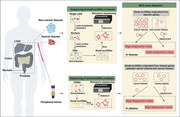

## 1 INTRODUCTION

Extrachromosomal circular DNA (eccDNA) is a circular DNA molecule that is independent of conventional chromosomes and exists widely in eukaryotes.^1^ The large species of eccDNAs are generally larger than 25 kb in size.^2^ Double minutes (DMs) are one of the most studied large species of eccDNAs, containing genes and regulatory elements that contribute to carcinogenesis and therapeutic resistance.^3^, ^4^, ^5^ The remaining 99% of eccDNAs, named as small eccDNAs, are shorter than 25 kb in length, and most of them are less than 1000 bp.^6^, ^7^, ^8^, ^9^ Recently, small eccDNAs have been reported to be related to microRNA expression and innate immunostimulatory activities.^8^, ^10^ However, the formation mechanism and function of small eccDNAs, especially their role in carcinogenesis, need to be further explored.

As small circular DNA molecules, small eccDNAs are promising biomarkers in cancer detection because they are structurally more stable than RNA and linear DNA.^11^, ^12^ However, few studies have analysed the relationship between small eccDNAs in tissues and those in the plasma in cancer patients (referred to as 'cancer plasma' hereafter). It is still unclear whether the small eccDNA profile in cancer plasma can represent that in cancer tissues.

Previous studies on small eccDNAs have mostly been based on the short‐read sequencer.^13^, ^14^ Small eccDNAs have to be fragmented during sample preparation, thus losing the full‐length information that is crucial for functional studies. More importantly, researchers often use bioinformatics tools and corresponding computational methods to indicate the presence of eccDNAs by discordant reads or split reads.^7^, ^12^, ^15^ However, in the absence of true full‐length information, algorithms must be used to calculate what the true eccDNAs look like and these calculated eccDNAs may be false‐positive detections.^7^ In particular, the identification of eccDNAs containing multiple fragments requires the calculation and matching of multiple junction positions.

To overcome these difficulties, we combined rolling circle amplification (RCA) with the nanopore sequencing platform to sequence full‐length small eccDNAs. We applied the novel workflow to multiple cancer types, with appropriately equal number of males and females, and described the differences of small eccDNAs between cancer and non‐cancer tissues. Another novelty of our study was the first comparison of small eccDNAs in the plasma and tissue collected from the same cancer patients, with an attempt to provide strong evidence for the study of stable cancer‐related small eccDNA biomarkers.

## 2 MATERIALS AND METHODS

### 2.1 Case recruitment

This study recruited 2 prostate cancer patients, 12 hepatocellular carcinoma patients, 15 colorectal cancer patients, 4 healthy volunteers and an independent validation cohort for the validation of cancer‐related biomarkers in plasma including 4 hepatocellular carcinoma patients, 3 colorectal cancer patients and 14 healthy volunteers (Supplementary Table S1–S2). All patients were treatment‐naïve and had no serious complications. According to the manufacturer's instructions, serum carcinoembryonic antigen (CEA) and carbohydrate antigen (CA)19‐9 levels were analysed by immunoassay (Roche Diagnostics).

### 2.2 Sample processing

Fresh tissue samples were washed with 1× phosphate‐buffered saline (PBS, Gibco) and cut into fragments using surgical scissors. Type I collagenase and type II collagenase (all Sigma) were mixed at 37°C for 10 min. 1 × TrypLE express enzyme (Gibco) was added to further digest at 37°C for 5 min. Red blood cells were removed with 1 × RBC Lysis buffer (Invitrogen). The cells were passed through a 70‐μm cell strainer (Corning) to obtain dissociated single cells. A total of 1 × 10^5^ cells were selected from each sample for small eccDNA extraction.

The 5 mL of peripheral blood samples were centrifuged at 2000 × *g* for 10 min at 4°C to obtain plasma. Plasma was centrifuged at 4600 × *g* for 10 min at 4°C to remove insoluble material.

### 2.3 Small eccDNA purification

For the extraction of small eccDNAs in tissues, cytoplasmic membrane lysis buffer (10 mM HEPES‐KOH, pH 7.9, 1.5 mM MgCl_2_, 10 mM DTT, 0.2 mM PMSF and 0.2% NP‐40) was added to the single‐cell precipitation and the mixture was incubated at 4°C for 15 min to destroy the cytoplasmic membrane (Supplementary Figure S1), and then nuclear precipitations were obtained by centrifuging at 1000 × *g* for 10 min at 4°C and washed twice by 1× PBS. Subsequently, nuclear membrane lysis buffer (5 mM Na_2_HPO_4_‐NaOH, pH 11.8, 0.75 mM MgCl_2_, 10 mM DTT, 0.2 mM PMSF and 2% NP‐40) was added to the precipitation and the mixture was incubated at 4°C for 20 min to destroy the nuclear membrane. Phenol: chloroform: isoamyl alcohol (25:24:1) (Sigma) was added to an equal volume of the solution for DNA purification. The reactants were shaken vigorously, allowed to stand at 4°C for 20 min, and centrifuged at 12 000 × *g* for 15 min at 4°C. The supernatant was pipetted and then mixed with 0.1 volumes of sodium acetate (3 M, pH 5.2) (Invitrogen) and 2 volumes of 95% ethanol. The mixture was mixed and kept at −20°C for 30 min and then centrifuged at 12 000 × *g* for 10 min at 4°C to obtain DNA pellets. The pellet was washed twice with 0.5 volumes of 70% ethanol. After drying, nuclease‐free water (Invitrogen) was added to dissolve the pellet.

For the extraction of small eccDNAs in plasma, cell‐free DNA was extracted using MagMAX™ cell‐free DNA isolation kit (ThermoFisher) from 2 mL of plasma samples.

For the purification of small eccDNAs in tissues and plasma, linear DNA was digested twice with 350 U of exonuclease III and 50 U of lambda exonuclease (all NEB) at 37°C for 2 h. When the crossing point (Cp) value of *COX5B* ^13^ (5′‐GGGCACCATTTTCCTTGATCAT‐3′ and 5′‐AGTCGCCTGCTCTTCATCAG‐3′) was greater than 40, linear DNA was indicated to be completely removed (Supplementary Figure S2 and Supplementary Table S3). SPRI beads (Beckman Coulter) with a size cutoff (2 beads:1 sample) were used to enrich small eccDNAs (> 50 bp).

### 2.4 Small eccDNA sequencing and identification

Small eccDNAs were added to 5 μL of 100 μM random hexamer primers. The samples were denatured at 95°C for 5 min, followed by annealing at 50°C for 15 s, 30°C for 15 s and 20°C for 10 min, and then hold on ice for 5 min. A reaction mix was added so that the final concentrations were 1 × phi29 DNA polymerase reaction buffer, 0.2 mg/mL BSA, 2 mM dNTP and 5 U of phi29 DNA polymerase (NEB). RCA was performed at 30°C for 24 h. The products were incubated with 10 U of T7 endonuclease I (NEB) at 37°C for 30 min. The small eccDNA sequencing library was prepared using the Ligation Sequencing Kit (SQK‐LSK109) and sequenced on the PromethION sequencer (R9.4.1, all Oxford Nanopore Technologies) (Supplementary Figure S3A–C). Raw reads with quality values of ≥10 were selected to map the human genome (*hg38*) using the minimap2 software. Small eccDNAs were generated from the raw reads (the number of tandem repeat sequences ≥ 2) using the eccDNA_RCA_nanopore software. Briefly, the workflow of the eccDNA_RCA_nanopore software was to map the raw reads to the human genome and then call the tandem repeat sequences not present in the human genome to generate small eccDNAs.

### 2.5 Polymerase chain reaction (PCR) for validation

Using the pre‐RCA DNA as a template, outward divergent primer sets (Supplementary Table 4–6) were designed to detect the target small eccDNAs by PCR. PCR products were loaded on the 2% agarose gel for electrophoresis. The base composition of PCR products was confirmed by Sanger sequencing.

### 2.6 Genomic distribution of small eccDNAs

The alignment result of small eccDNAs with the *hg38* reference genome was obtained by the minimap2 package (V2.18). After collapsing small eccDNA that appeared many times in a single read into one small eccDNA, the coverage of small eccDNA fragments in each window (1 000 000 bp) of the genome was obtained using the bedtools package (V2.30.0), with the alignment result as input. After normalising coverage, the median distribution of small eccDNA fragments across each chromosome was plotted by the RIdeogram package (V0.2.2).

### 2.7 Junctional motifs of small eccDNAs

The junction position of small eccDNAs was defined as the site where two ends of genomic sequences ligated to generate small eccDNAs. To explore the motif patterns of junction position, the MEME package (V5.3.0) was used to scan the base composition from 50 bp upstream to 50 bp downstream of the start and end positions of the genomic fragment corresponding to each small eccDNA.

### 2.8 Genomic annotation of small eccDNAs, GENE ONTOLOGY (GO) analysis and Kyoto Encyclopedia of Genes and Genomes (KEGG) pathway enrichment analysis of small eccDNA‐associated genes

Small eccDNA was mapped to the genomic features (*hg38*) by the annotatePeaks function in the HOMER package (V4.11). The genes corresponding to small eccDNAs were defined as small eccDNA‐associated genes. The difference in the proportion of these small eccDNAs in paired samples was assessed by DESeq2 by the criteria of at least a twofold change and *padj* < .05. GO and KEGG pathway enrichment analyses of small eccDNA‐associated genes were assessed by DAVID software. *P* < .05 was considered to indicate significance.

### 2.9 Evaluation of small eccDNAs as biomarkers for cancer diagnosis

The proportion of small eccDNAs originated from a specific gene was used to predict the presence of cancer based on receiver operating characteristic (ROC) curves.

$$=\frac{\begin{array}{c} \mathrm{The}\;\mathrm{proportion}\;\mathrm{of}\;\mathrm{small}\;\mathrm{eccDNAs}\;\mathrm{originated}\;\mathrm{from}\;a\;\mathrm{specific}\;\mathrm{gene}\\ \mathrm{the}\;\mathrm{number}\;\mathrm{of}\;\mathrm{small}\;\mathrm{eccDNAs}\;\mathrm{originated}\mathrm{from}\;a\;\mathrm{specific}\;\mathrm{gene}\;\left(\mathrm{including}\;\mathrm{exon}\;\mathrm{and}\;\mathrm{intron}\right) \end{array}}{\mathrm{the}\;\mathrm{number}\;\mathrm{of}\;\mathrm{annotations}\;\mathrm{by}\;\mathrm{the}\;\mathrm{HOMER}\;\mathrm{software}}.$$

The proportion of small eccDNAs originated from specific genes was combined with CEA/CA19‐9 levels to construct a logistic regression model and calculate fitted probabilities, and then the fitted probabilities were used to predict the presence of cancer based on ROC curves. Using the boot.roc algorithm in the fbroc R package, we generated ROC curves by bootstrapping for 2000 replicates.^16^ The area under the ROC curve (AUC) was calculated. Cut‐off points were selected using Youden index.^17^

### 2.10 Statistical analyses

Paired samples conforming to the normal distribution and those not conforming to the normal distribution were compared by two‐tailed paired *t*‐test and Wilcoxon signed‐rank test, respectively, by SPSS software (V25.0). Statistical significance was defined as *P* < 0.05.

## 3 RESULTS

### 3.1 An efficient and cost‐efficient method to purify small eccDNAs from tissues

**FIGURE 1:**
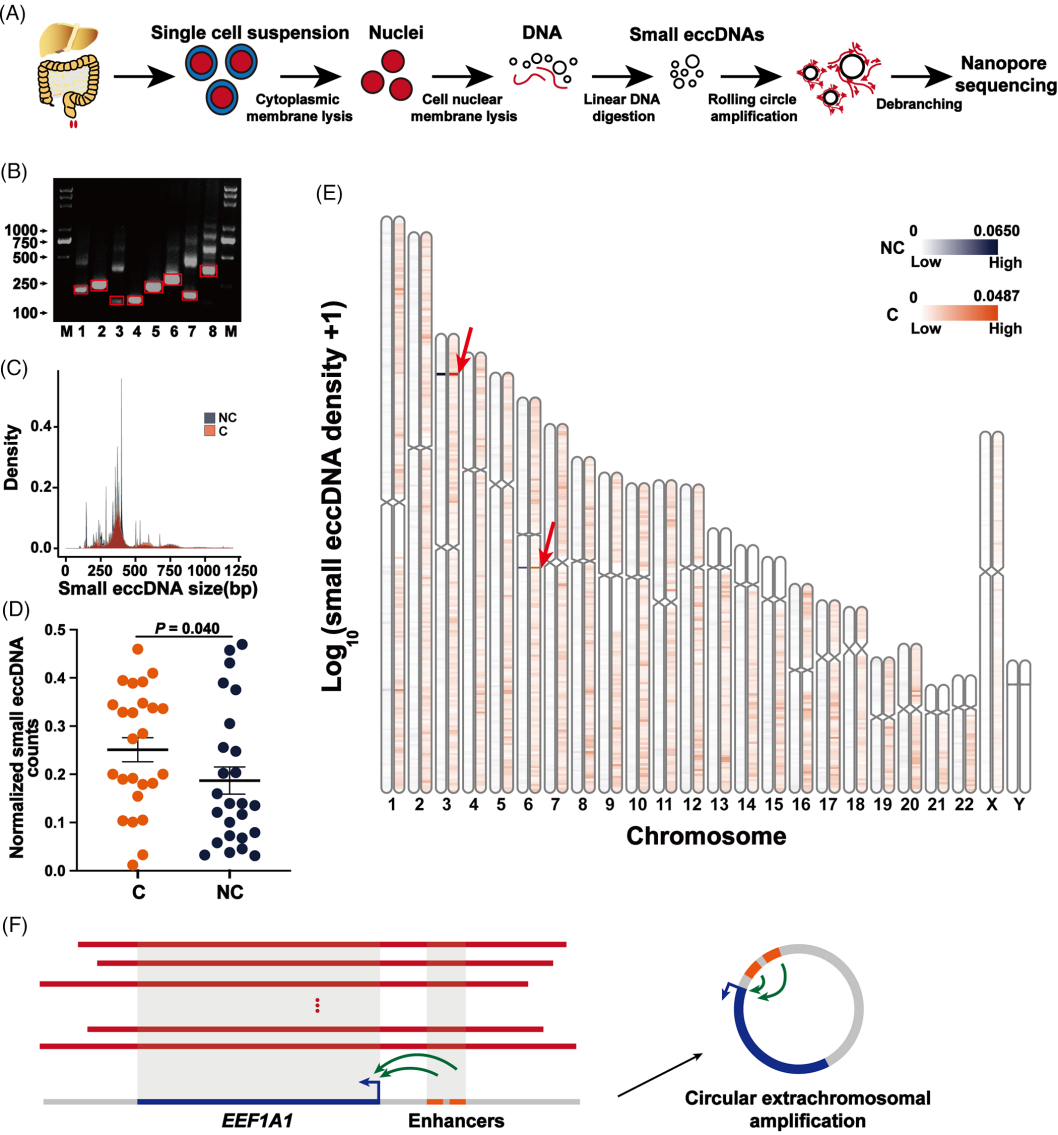
The general characteristics of small eccDNAs in non‐cancer tissues and cancer tissues. (A) Workflow of small eccDNA extraction, purification and sequencing. (B) Validation of eight small eccDNAs from tissues by PCR analysis. The DNA bands in the red rectangular box are PCR products used for Sanger sequencing. Lane M: DNA marker; lane 1: Chr3:181166649‐181166872; lane 2: Chr4:175249993‐175250381; lane 3: Chr13:42525227‐42525567; lane 4: Chr5:149499532‐149499733; lane 5: Chr4:103896308‐103896614; lane 6: Chr1:202005207‐202005611; lane 7: Chr16:54051385‐54051620; Chr8: Chr10:109212714‐109213124. (C) The size distribution of small eccDNAs (less than 1200 bp) in non‐cancer (NC) and cancer (C) tissues. (D) The normalised amount of small eccDNAs detected in each sample from non‐cancer (NC) and cancer (C) tissues. To make every sample comparable, the value of the small eccDNA count was divided by the number of reads mapped to the human genome (*hg38*) in the sample, which we termed as 'normalised small eccDNA counts'. After normality transformation (arcsine square root transformation), the significant difference between the two groups was analysed by two‐tailed paired *t*‐test. (E) Overall chromosomal distribution of small eccDNAs across the genome in non‐cancer (NC) and cancer (C) tissues. (F) The structure of small eccDNA originated from the full‐length eukaryotic translation elongation factor 1 alpha 1 *(EEF1A1)* gene.

To enrich nuclear small eccDNAs efficiently and cheaply, a customized approach for eccDNA purification was modified (Figure 1A).^6^ Firstly, the cell plasma membrane was directly disrupted by a hypotonic buffer with a small amount of NP‐40, thus simplifying the conventional nuclear extraction process.^6^, ^18^ Mitochondrial DNA was the most abundant circular DNA in the cytoplasm. In all tissue samples, the median mitochondrial DNA contamination in raw reads was only 0.0037% (Supplementary Figure S3D), which is lower than the digestion results (0.2–0.3%) of the combination of *Not*I and Plasmid‐Safe ATP‐dependent DNase,^7^, ^19^ indicating successful nucleic acid removal in the cytoplasm. Next, an alkaline buffer combined with a large amount of NP‐40 was used to break the nuclear membranes, instead of unbuffered sodium hydroxide which might cause irreversible denaturation or breakage of DNA circles.^10^, ^20^ Then, Exonuclease III and lambda exonuclease were utilised to digest linear DNA. Finally, the modified tissue small eccDNA purification workflow combined with RCA was used to perform nanopore sequencing on 50 tissue samples (Figure 1A and Supplementary Table S1).

To determine the reliability of small eccDNAs detected by our method, eight small eccDNAs were randomly selected for outward PCR and Sanger sequencing to validate the sequences at and around the junction positions. The results were consistent with those detected by our method (Figure 1B and Supplementary Table S4). In addition, we selected a plasmid (4842 bp) as a positive control and the linear full‐length PCR product of this plasmid (4842 bp) as a negative control to further validate our workflow starting from adding nuclear membrane lysis buffer. The qPCR results before and after exonuclease digestion, and after RCA showed the successful removal of linear DNA and the enrichment of circular DNA (Supplementary Figure S4A). Next, we observed that small eccDNAs detected in the positive control were able to align with the full‐length sequence of the plasmid (Supplementary Figure S4B). No small eccDNA was generated in the negative control.

### 3.2 General characteristics of small eccDNAs in cancer tissues

Small eccDNAs in cancer and non‐cancer tissues showed comparable size distributions (Figure 1C), displaying a main peak at about 380 bp and some periodic peaks at about 10 bp. Nearly 99% of small eccDNAs were shorter than 1200 bp. This size distribution was consistent with previous human cancer cell line data generated from eccDNA purification and RCA.^10^ Interestingly, the size and number of small eccDNAs in cancer tissues (median number = 239 835) were significantly larger than those in paired non‐cancer tissues (median number = 72 849) (Supplementary Figure S5 and Figure 1D). Chromosomes (Chr) 4, 7, 8 and 11 were differentially represented in small eccDNA frequencies between cancer (median = 5.569%, 5.235%, 4.994% and 4.234%, respectively) and non‐cancer tissues (median = 3.867%, 3.839%, 2.471% and 2.448%, respectively) (Supplementary Figure S6). Small eccDNAs were enriched in the specific positions of Chr3 (Chr3:17 000 000–18 000 000) and Chr6 (Chr6:73 000 000–74 000 000) (Figure 1E). Notably, the distribution density of ChrY was relatively lower, which may be due to the lack of genomic recombination and functional genes,^21^, ^22^ and another possible explanation is that the poorer genomic annotation and more repetitive DNA in ChrY make it harder to map small eccDNAs to this region. In addition, small eccDNAs in two colorectal cancer tissues and one non‐cancer tissue were found to originate from the full‐length eukaryotic translation elongation factor 1 alpha 1 (*EEF1A1*) gene and two enhancers of *EEF1A1* (Figure 1F) (The different lengths of each small eccDNA may be due to their origins in apoptotic DNA fragments^10^) and may function independently in the development of colorectal cancer.^23^

Further analysis focused on small eccDNAs composed of two fragments (2f small eccDNAs). Interestingly, compared with non‐cancer tissues, cancer tissues had more 2f small eccDNA events on each chromosome, and the fragments contained in the 2f small eccDNA events were more random (Supplementary Figure S7). This may be due to more frequent chromosomal spatial contacts in cancer tissues,^24^ which favour the direct generation of 2f small eccDNAs, or because the fusion evolution of small eccDNAs containing a fragment is more active in cancer tissues.^25^

### 3.3 Specific junctional motifs and genomic origin of small eccDNAs in cancer tissues

**FIGURE 2:**
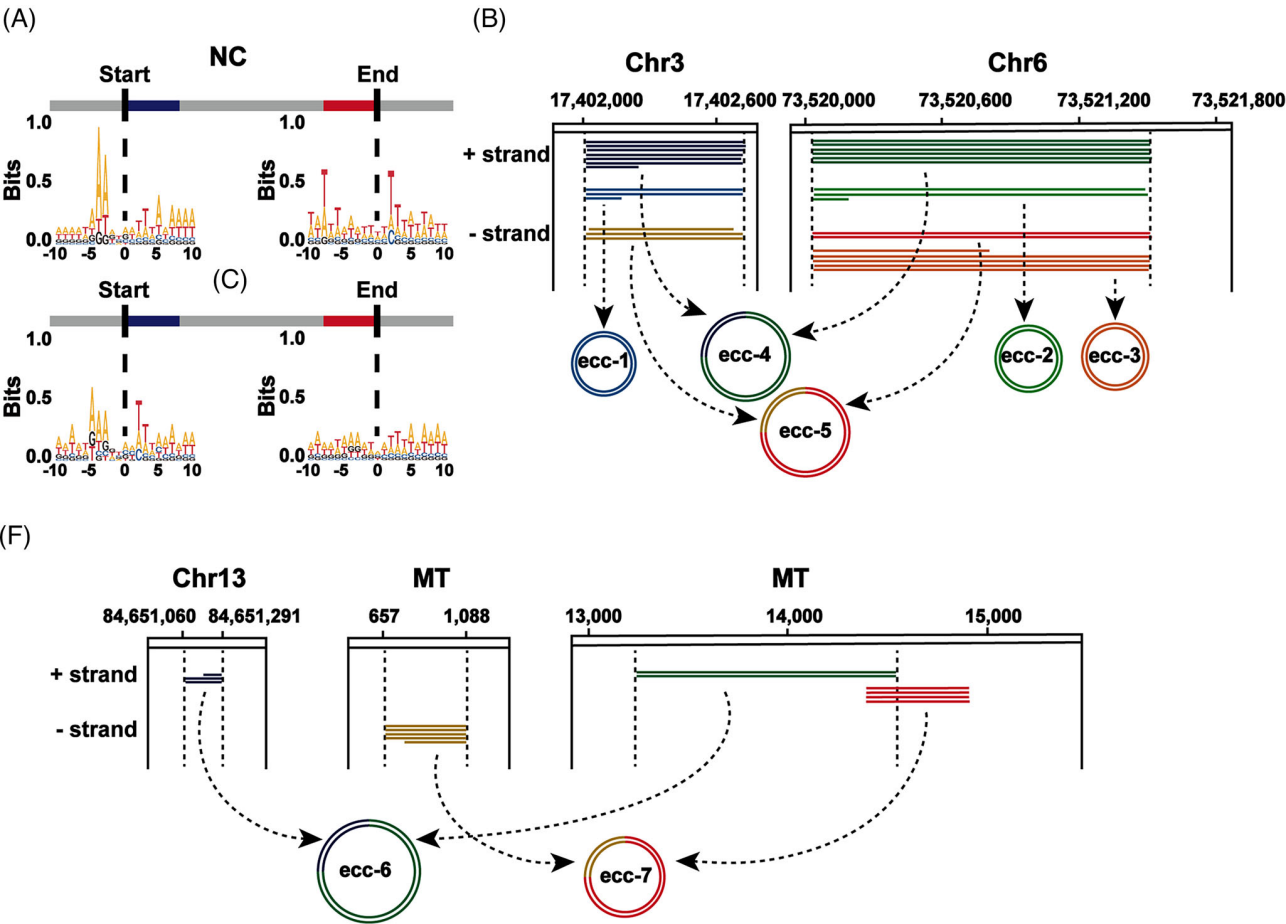
Biogenesis mechanism of small eccDNAs in cancer tissues. (A) Nucleotide motif sequences flanking the start and end positions of small eccDNAs in non‐cancer (NC) and cancer (C) tissues. (B) Integrative Genomics Viewer (IGV) alignments showing small eccDNAs from two genomic loci on chromosomes 3 and 6 in the same tissue sample (C52) as an example. Individual horizontal bars in the same colour represent subreads from a unique long read that are originated from the same genomic locus. Different colours represent different raw reads, and the number of lines represents the number of tandem repeats. ecc‐1, ecc‐2 and ecc‐3 are single‐fragment circles. ecc‐4/ecc‐5 consists of ecc‐1 and ecc‐2/ecc‐3. Chr, chromosome. (C) IGV alignments showing small eccDNA examples from mitochondrial and genomic loci. MT, mitochondria.

Next, we explored the nucleotide motif sequence of the junction position, which might contribute to elucidating the generation mechanisms of small eccDNAs.^26^ The motif sequences of the junction positions in small eccDNAs showed the preferential use of A and T bases (Figure 2A). In non‐cancer tissues, the upstream sequence of the start position (−7 to −3 bp) and the downstream sequence of the end position (2–6 bp) of small eccDNAs showed complementary trends that contributed to the detachment of DNA fragments from chromosomes to generate small eccDNAs through the microhomology‐mediated end joining pathway.^27^ However, this phenomenon was not found in cancer tissues, suggesting that the formation mechanism of small eccDNAs in cancer tissues may be different from that in non‐cancer tissues.

Surprisingly, in both cancer and non‐cancer tissues, we observed a common phenomenon that some fragments not only were involved in the production of small eccDNA containing a single fragment but also participated in the generation of 2f small eccDNAs (Figure 2B), suggesting the evolution of small eccDNAs by fusion after initial formation.^25^ The enlargement of small eccDNAs was thought to occur through the breakage‑fusion‑bridge (BFB) mechanism.^2^ Moreover, the mitochondrial DNA fragments formed small eccDNAs not only by themselves but also together with the chromosomal fragments (Figure 2C). Since there was little spatial contact between mitochondrial DNA and chromosomes, there might be fragments from chromosomes or mitochondria crossing the nuclear membrane, most likely in the form of small eccDNAs.

**FIGURE 3:**
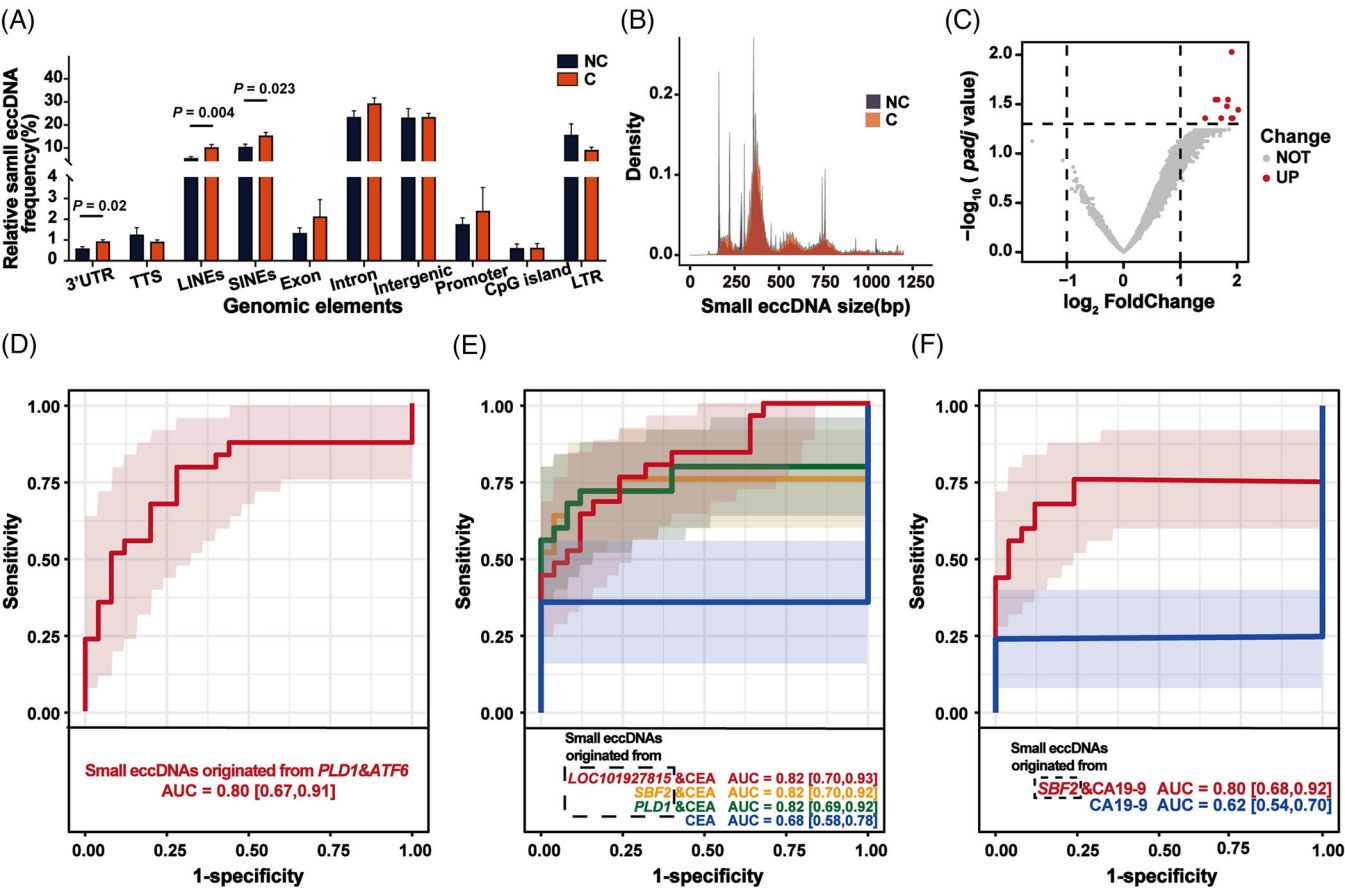
Genomic functional annotation of small eccDNAs in cancer tissues. (A) Genomic distribution of small eccDNAs in non‐cancer (NC) and cancer (C) tissues. UTR, untranslated region; TTS, transcription start site; LINEs, long interspersed nuclear elements; SINEs, short interspersed nuclear elements; LTR, long terminal repeat. * There are significant differences. (B) The size distribution of small eccDNAs (less than 1200 bp) originated from the long interspersed element‐1 (LINE‐1) region in non‐cancer (NC) and cancer (C) tissues. (C) Volcano map of the difference in the proportion of small eccDNAs originated from specific genes between non‐cancer tissues and cancer tissues. (D) The multi‐cancer diagnostic value of the combination of the proportion of small eccDNAs originated from *PLD1* and *ATF6* (*P* < 0.05). *PLD1*, phospholipase D1; *ATF6*, activating transcription factor 6; AUC, the area under the ROC curve. (E–F) Great multi‐cancer diagnostic value of the combination of the proportion of small eccDNAs originated from specific genes and CEA/CA19‐9 levels (all AUC ≥ 0.8 and *P* < 0.05). CEA, carcinoembryonic antigen; *SBF2*, SET Binding Factor 2; *ETV6*, ETS variant transcription factor 6; CA19‐9, carbohydrate antigen 19‐9.

Based on the annotation results of HOMER software, we found that the proportions of small eccDNAs enriched with 3′UTR, long interspersed nuclear elements (LINEs) and short interspersed nuclear elements (SINEs) regions in cancer tissues were significantly larger than that in paired non‐cancer tissues (Figure 3A), which may be due to many genomic alterations in cancer tissues.^28^ Small eccDNAs were enriched with LINEs, SINEs and long terminal repeat (LTR) regions, suggesting that they may have functions similar to those of retrotransposons. Considering that long interspersed element‐1 (LINE‐1) was an autonomous retrotransposon in the human genome, we further analysed the size distribution of small eccDNAs carrying LINE‐1 elements and found that small eccDNAs were 200–800 bp in size (Figure 3B). We then observed that these small eccDNAs were composed of intergenic or intron regions of LINE‐1, indicating that they are not capable of autonomous transposition.

### 3.4 High multi‐cancer diagnostic value of small eccDNAs originated from specific genes

We collected small eccDNAs originated from exon and intron regions, and found that small eccDNAs originated from 10 genes were more common in cancer tissues than in paired non‐cancer tissues (Figure 3C). We then investigated the multi‐cancer diagnostic value of small eccDNAs originated from these 10 genes as a proportion of all small eccDNA annotations (Supplementary Figure S8 and Supplementary Table S7). Strikingly, the combination of small eccDNAs originated from phospholipase D1 (*PLD1*) and activating transcription factor 6 (*ATF6*) had great multi‐cancer diagnostic value (Figure 3D and Supplementary Table S7). In the processes of carcinogenesis and metastasis*, PLD1* acts as a downstream effector of various cell‐surface receptors to trigger and regulate the propagation of intracellular signals in various cancers.^29^ *ATF6* serves as an important regulator of organogenesis and tissue homeostasis, and aberrant *ATF6* activity may promote the pathogenesis of various cancers.^30^

Given that CEA and CA19‐9 are directly secreted by the tumour, we hypothesised that the readouts provided by CEA/CA19‐9 serum levels and by small eccDNAs were complementary. Indeed, combined with differential small eccDNAs between cancer and non‐cancer tissues, the diagnostic accuracy of CEA/CA19‐9 increased from AUC = 0.68/0.62 to ≥ 0.80 (Figure 3E–F and Supplementary Table S7).

### 3.5 The general characteristics of small eccDNAs in cancer plasma were different from those in cancer tissues

**FIGURE 4:**
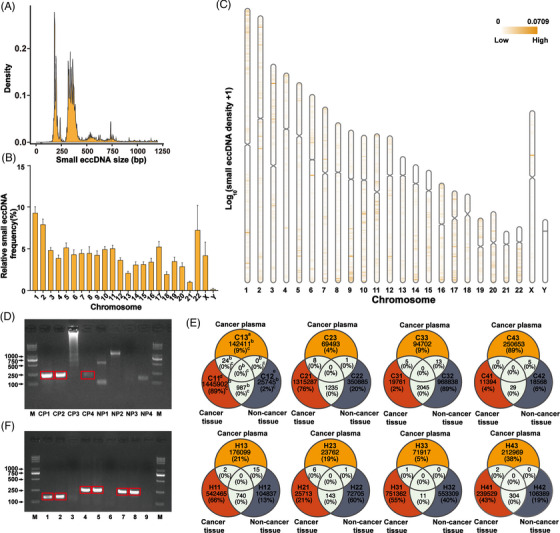
The general characteristics of small eccDNAs in cancer plasma. (A) The size distribution of small eccDNAs (less than 1200 bp) detected in cancer plasma. (B) The distribution of small eccDNAs detected in cancer plasma on each chromosome. (C) Overall chromosomal distribution of small eccDNAs across the genome in cancer plasma. (D) Detection of a specific small eccDNA (Chr6:46819468‐46819807) in four cancer plasma and four normal plasma by PCR analysis. The DNA bands in the red rectangular box are PCR products used for Sanger sequencing. Lane M: DNA marker. CP, cancer plasma; NP, normal plasma. CP1 and CP2 were plasma samples from patients with colorectal cancer. CP3 and CP4 were plasma samples from patients with hepatocellular carcinoma. NP1–NP4 were plasma samples from healthy people. (E) Veen plots of small eccDNAs detected in cancer plasma, cancer tissues and non‐cancer tissues from the same patient. The figure on the upper left is an example of the details, and all other figures are also applicable: a/d/e, the sample names; C11, a cancer tissue sample from patient C1; C12, a non‐cancer tissue sample from patient C1; C13, a cancer plasma sample from patient C1; b, the number of small eccDNAs detected in each sample; c, the percentage of small eccDNAs detected in this sample to the sum of small eccDNAs detected in cancer plasma, cancer tissues and non‐cancer tissues of the same patient. (F) Validation of the small eccDNA originated from Chr17:30186596‐30186933 (lanes 1−3), Chr17:64682690‐64683063 (lanes 4−6) and Chr8:100632795‐100633127 (lanes 7−9). The DNA bands in the red rectangular box are PCR products used for Sanger sequencing. Lane M: DNA marker; lanes 1, 4 and 7: validation in a cancer tissue sample (C11); lanes 2, 5 and 8: validation in a cancer plasma sample (C13); lanes 3, 6 and 9: validation in a non‐cancer tissue sample (C12).

Four plasma samples from patients with colorectal cancer and four plasma samples from patients with hepatocellular carcinoma, which were paired with these tissues, were randomly selected for small eccDNA sequencing. We identified 23 769 to 916 854 small eccDNAs in these plasma samples. Unlike cancer tissues (Figure 1C), the size of small eccDNAs in cancer plasma displayed bimodal distribution at ∼190 and ∼350 bp (Figure 4A). The size of small eccDNAs in cancer plasma was significantly less than that in cancer tissues (*P* < 2.2e‐16), which may be due to the digestion of plasma nucleases and the cell phagocytosis.^31^, ^32^ Unlike cancer tissues (Supplementary Figure S6), small eccDNAs in cancer plasma were mainly from Chr1 and Chr2 (Figure 4B). As shown in Figure 4C, the small eccDNAs in cancer plasma were not enriched in the specific positions of chromosomes (Chr3:17 000 000–18 000 000 and Chr6:73 000 000–74 000 000) detected in cancer tissues (Figure 1E).

Some small eccDNAs not identified in non‐cancer tissues were shared in these cancer plasma samples. Of these shared small eccDNAs originated from exons, one small eccDNA (Chr6:46 819 468–46 819 807) originated from meprin a subunit alpha (*MEP1A*) was selected and validated in plasma samples from eight other subjects (Figure 4D and Supplementary Table S5). *MEP1A* is not only involved in the progression of hepatocellular carcinoma and colorectal cancer but was also used as a prognostic biomarker.^33^, ^34^ The results showed that this small eccDNA was detectable in three cancer plasmas but not in normal plasma, suggesting the potential of these shared small eccDNAs to be used as novel cancer‐specific biomarkers.

Next, we compared the sequence similarity of small eccDNAs between cancer plasma and the paired tissues (Figure 4E). There were some shared small eccDNAs between cancer tissues and the paired cancer plasma, but not detected in the paired non‐cancer tissues. And we randomly selected three small eccDNAs for validation in cancer tissue, non‐cancer tissue and cancer plasma samples (Figure 4F and Supplementary Table S6). These shared small eccDNAs were found to be individual‐specific, which could be used as biomarkers to track the effects of individualised cancer treatments.

### 3.6 Different genomic annotation but similar junctional motifs of small eccDNAs between cancer plasma and cancer tissues

**FIGURE 5:**
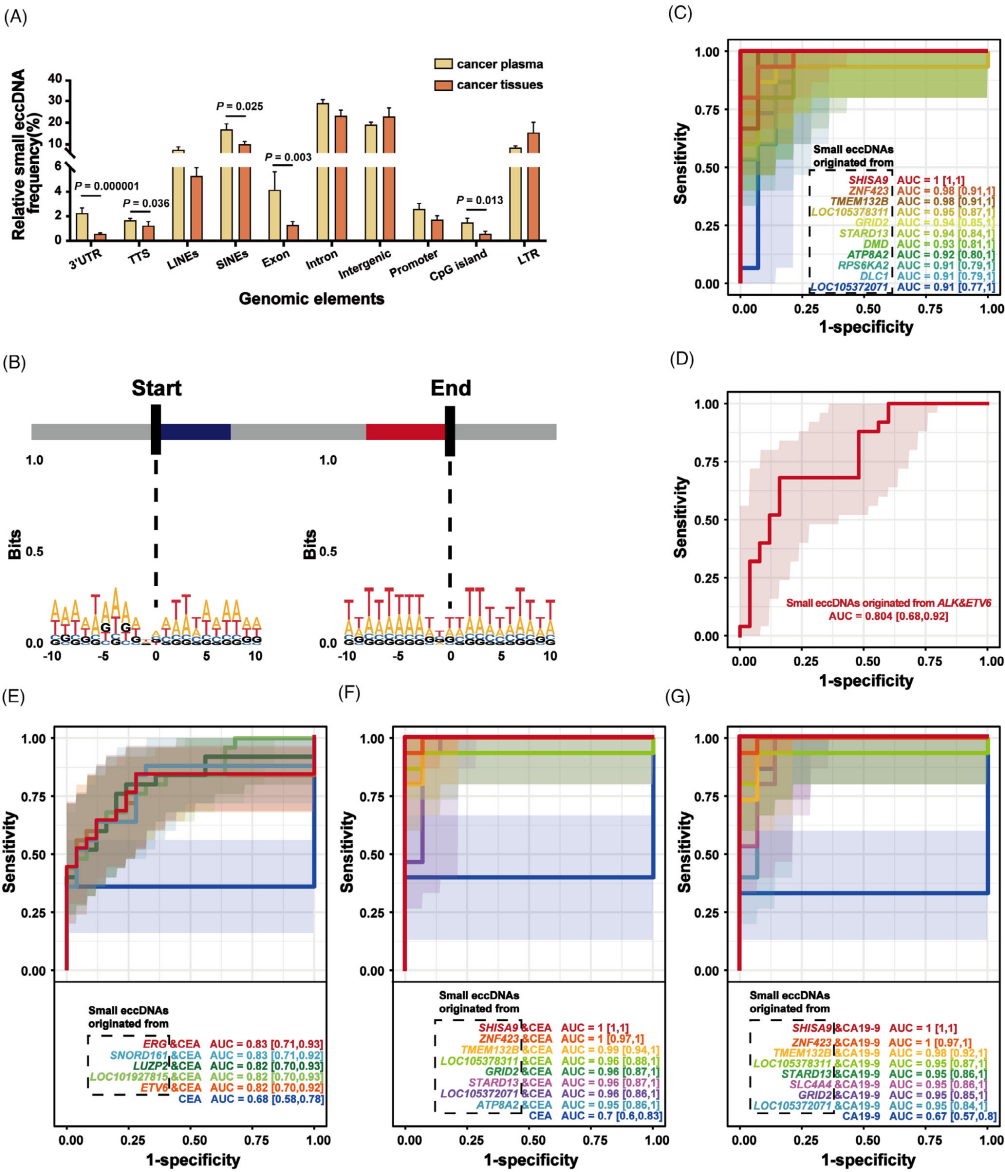
Motif analysis of small eccDNA junctions in cancer plasma and multi‐cancer diagnostic value of small eccDNAs. (A) Genomic distributions of small eccDNAs in cancer plasma and cancer tissues. UTR, untranslated region; TTS, transcription start site; LINEs, long interspersed nuclear elements; SINEs, short interspersed nuclear elements; LTR, long terminal repeat. * There are significant differences. (B) Nucleotide motif sequences flanking the start and end positions of small eccDNA junctions in cancer plasma. (C) The multi‐cancer diagnostic value in plasma of the proportion of small eccDNAs originated from some genes (all AUC > .9 and *P* < .05). *ZNF423*, zinc finger protein 423; *SHISA9*, shisa family member 9; *TMEM132B*, transmembrane protein 132B; *GRID2*, glutamate ionotropic receptor delta type subunit 2; *STARD13*, StAR related lipid transfer domain containing 13; *DMD*, dystrophin; *DLC1*, DLC1 Rho GTPase activating protein; *LMCD1‐AS1*, LMCD1 antisense RNA 1; *RPS6KA2*, ribosomal protein S6 kinase A2; *SLC4A4*, solute carrier family 4 member 4; *ATP8A2*, ATPase phospholipid transporting 8A2; AUC, the area under the ROC curve. (D) The multi‐cancer diagnostic value in tissues of the proportion of small eccDNAs originated from *ALK* and *ETV6* (*P* < 0.05). *ALK*, ALK receptor tyrosine kinase; *ETV6*, ETS variant transcription factor 6. (E) Great multi‐cancer diagnostic value in tissues of the combination of the proportion of small eccDNAs originated from some specific genes and CEA (all AUC > .8 and *P* < .05). *ERG*, ETS transcription factor ERG; CEA, carcinoembryonic antigen; *SNORD161*, small nucleolar RNA, C/D box 161; *LUZP2*, leucine zipper protein 2. (F–G) Great multi‐cancer diagnostic value in plasma of the combination of the proportion of small eccDNAs originated from some specific genes and CEA/CA19‐9 (all AUC ≥ .95 and *P* < .05). CA19‐9, carbohydrate antigen 19‐9.

The proportions of small eccDNAs originated from 3′UTR, TSS, SINEs, exon and CpG island regions in cancer plasma were significantly higher than those in cancer tissues (Figure 5A), indicating that small eccDNAs between cancer plasma and cancer tissues have different preferences of genomic origin. We then focused on the small eccDNAs originated from exons and found that two small eccDNAs were significantly enriched in cancer plasma, including one originated from *MEP1A* and the other originated from myosin 18b (*MYO18B*) (Supplementary Figure S9). *MEP1A* has been considered as a prognostic biomarker and found to promote proliferation and invasion of various cancers.^33^, ^34^, ^35^ *MYO18B* serves as a candidate tumour suppressor gene whose activity is involved in the progression of several cancers.^36^, ^37^, ^38^

To explore the formation mechanism of small eccDNAs in cancer plasma, we analysed the nucleotide motif patterns of the junction position in small eccDNAs. Similar to that in cancer tissues (Figure 2A), the motif sequence of the junction position in small eccDNAs showed a preference for A and T bases in cancer plasma, and the start and end positions of the junctions were flanked by a pair of high‐frequency nucleotide segments (−5 to −3 bp and 2–4 bp) with 4‐bp 'spacers' in between (Figure 5B), indicating that small eccDNAs in cancer tissues and cancer plasma have similar formation mechanisms.

### 3.7 Cancer plasma harboured characteristic small eccDNAs consistent with cancer tissues, with great multi‐cancer diagnostic value

We identified 72 shared small eccDNA‐associated genes between cancer plasma and cancer tissues, including some cancer‐related genes, such as ALK receptor tyrosine kinase (*ALK*), ETS transcription factor ERG (*ERG*), ETS variant transcription factor 6 (*ETV6*) and casein kinase 2 alpha 3 (*CSNK2A3*).^39^ Moreover, KEGG enrichment analysis of those shared small eccDNA‐associated genes was enriched in some cancer‐related pathways (Supplementary Figure S10).

Next, 7 cancer plasma samples and 14 normal plasma samples were collected as an independent validation cohort (Supplementary Table S2) for biomarker analysis. The proportion of small eccDNAs originated from these shared genes showed high multi‐cancer diagnostic value in tissues (Supplementary Figure S11 and Supplementary Table S7) and plasma (Figure 5C and Supplementary Table S8). Importantly, the combination of small eccDNAs originated from *ETV6* (cut‐off point: 0.000019) and *ALK* (cut‐off point: 0.000022) showed great multi‐cancer diagnostic value in tissues (Figure 5D and Supplementary Table S7). Moreover, the combination of these cut‐off points showed high diagnostic sensitivity (100%) in these eight cancer plasma samples paired with cancer tissues (Supplementary Table S8). Next, we applied the combined cut‐off point to plasma samples from the independent validation cohort and found high positive predictive value (100%) and negative predictive value (82.35%), suggesting its potential as a biomarker for non‐invasive multi‐cancer diagnostics (Supplementary Table S8).

Meanwhile, by combining with small eccDNAs originated from these shared genes, the accuracy of multi‐cancer prediction of CEA/CA19‐9 levels has been improved to AUC > 0.80 and 0.95 in tissue or plasma, respectively (Figure 5E–G and Supplementary Table S7–S8).

## 4 DISCUSSION

Small eccDNAs have been implicated in several important biological processes, including the promotion of innate immune responses,^10^ the generation of short regulatory RNAs to regulate gene expression independent of canonical promoters^8^ and as by‐products of apoptosis.^40^ However, the high cost of small eccDNA sequencing has limited further investigation. Our study provides a workflow that can reduce the cost to approximately $200–$300 per sample. More importantly, our workflow can directly obtain the full‐length sequences of small eccDNAs and therefore is more advantageous to accurately identify small eccDNAs composed of multiple fragments than other methods that use assembly algorithms to obtain matching junction positions and fragments.^7^, ^12^, ^15^

Unlike the bulk sequencing based on the same amount of genomic DNA,^41^ our study truly reflected differences in small eccDNAs within the same number of cells in cancer and non‐cancer tissues. Compared to whole‐cell small eccDNA sequencing,^42^ we used a hypotonic buffer with a small amount of NP‐40 to refine the object into two‐cell compartments. Although our focus was on small eccDNAs in the nuclei, other scientists can use our workflow to investigate small eccDNAs in the cytoplasm. Considering that the combination^7^, ^19^, ^42^ of restriction endonuclease and Plasmid‐Safe ATP‐dependent DNase for removing mitochondrial DNA may result in the loss of some small eccDNA information, our pipeline takes the step of separating cytoplasmic and nuclear fractions to successfully remove mitochondrial DNA, which not only avoids the digestion of small eccDNAs but also simplifies the experimental process and saves costs. This new workflow is easy to operate and standardise and can be performed with common laboratory equipment. Thus, it has the potential to deepen research on small eccDNAs and be integrated into the automated pipeline for large‐scale commercial applications.

One focus of our study was to profile the different characteristics of small eccDNAs in cancer tissues and non‐cancer tissues. There were only a small number of small eccDNAs in some cancer and non‐cancer tissues, which may be due to the high individual variation in cancer patients. Compared with that in non‐cancer tissues, the number of small eccDNAs in cancer tissues was larger, which might be caused by pervasive chromosomal instability in cancer.^43^ Small eccDNAs have high innate immunostimulatory activity,^10^ so the increase in small eccDNA content may be beneficial to promote anti‐tumour immune responses. Meanwhile, the formation mechanism of small eccDNAs in cancer tissues was different from that in non‐cancer tissues, and the regularity of junction position was weaker, which was likely due to the stronger chromosome instability in cancer tissues and the changes in the tumour microenvironment, although the underlying mechanisms warrant further research.

We found that small eccDNAs were primarily originated from retrotransposons and speculated that it can have functions similar to retrotransposons (e.g. leading to genomic instability and contributing to cancer development^44^). The methylation modification information of these small eccDNAs can be supplemented by combining tagmentation with 5‐mC‐Tn5 transposomes and short‐read sequencing technology.^45^, ^46^, ^47^

Small eccDNAs can move across the nuclear membrane and the shared small eccDNAs between cancer tissues and plasma are enriched in some cancer‐related pathways; thus, they have the potential to dynamically regulate various life activities in multiple cellular compartments, especially the occurrence and progression of cancer. We observed that small eccDNAs can evolve by fusion of small eccDNA molecules, which is consistent with the fact that eccDNAs can gradually increase in size by fusing with other eccDNAs.^9^ Therefore, we speculate that small eccDNAs may serve as a resource reserve pool of megabase‐sized eccDNAs to play a role in driving cancer progression.

Less than 0.01% of small eccDNAs were shared between cancer tissues and cancer plasma, which may be because both normal and cancer tissues contain small eccDNAs and can release them into the circulation.^12^, ^48^ Importantly, most of these shared small eccDNAs could not be detected in non‐cancer tissues. Therefore, these shared small eccDNAs may have the potential as non‐invasive biomarkers for multi‐cancer diagnostics. Indeed, great multi‐cancer diagnostic value in tissues was observed in small eccDNAs originated from some specific genes, especially the combination of *PLD1* and *ATF6*, or *ETV6* and *ALK*. More strikingly, the combination of *ETV6* and *ALK* can also be extrapolated to multi‐cancer diagnostics in plasma and was validated in an independent cohort. Besides, a large increase in diagnostic accuracy of CEA/CA19‐9 was observed in the combination of small eccDNAs originated from some specific genes in plasma, and with potential towards cost‐effective non‐invasive multi‐cancer screening and monitoring. In the future, we will expand the sample size to validate this finding and look for more cancer‐specific small eccDNAs.

Taken together, the comprehensive characterisation profiling of small eccDNAs for cancer patients in our study provides abundant human data for the future study of the mechanism of small eccDNAs in carcinogenesis. The small eccDNA data from cancerous and para‐cancerous tissues, coupled with those from the remote peripheral blood, can serve as a rich resource for a deeper understanding of the transport and flow of small eccDNAs, and pave the way for rational development of cancer‐specific small eccDNA biomarkers.

## AUTHOR CONTRIBUTIONS

All authors confirmed they have contributed to the intellectual content of this paper and have met the following four requirements: (a) significant contributions to the conception and design, acquisition of data or analysis and interpretation of data; (b) drafting or revising the article for intellectual content; (c) final approval of the published article and (d) agreement to be accountable for all aspects of the article, thus ensuring that questions related to the accuracy or integrity of any part of the article are appropriately investigated and resolved.

Conceptualisation & methodology, X.M.L, formal analysis, L.L.Z, writing‐original draft, X.M.L. and L.L.Z., data curation, Z.F.Z., resources, J.C. and Q.A., validation, X.M.L. and H.X.L., visualisation, X.M.L., G.Y.S., W.H., Y.F.L. and F.S., project administration, W.Z.J., supervision, L.H.Z, G.Z. and F.X.

## FUNDING INFORMATION

This work was supported by the National High Level Hospital Clinical Research Fund (Grant BJ‐2023‐077) and the CAMS Innovation Fund for Medical Sciences (2021‐I2M‐1‐050).

## CONFLICT OF INTEREST STATEMENT

The authors declare no conflicts of interest.

## ETHICS APPROVAL AND CONSENT TO PARTICIPATE

The study was approved by the Beijing Hospital Ethics Committee (Agreement Number: 2017BJYYEC‐108‐05). All the subjects provided written informed consent.

## HUMAN GENES

*EEF1A1*, eukaryotic translation elongation factor 1 alpha 1; *SBF2*, SET Binding Factor 2; *ARHGEF28*, Rho guanine nucleotide exchange factor 28; *ATF6*, activating transcription factor 6; *PLD1*, phospholipase D1; *ETV6*, ETS variant transcription factor 6; *MEP1A*, meprin a subunit alpha; *MYO18B*, myosin 18B; *ALK*, ALK receptor tyrosine kinase; *ERG*, ETS transcription factor ERG; *SYNE1‐AS1*, SYNE1 antisense RNA 1; *CSNK2A3*, casein kinase 2 alpha 3; *SNORD161*, small nucleolar RNA, C/D box 161; *C10orf90*, chromosome 10 open reading frame 90; *LUZP2*, leucine zipper protein 2; *ZNF423*, zinc finger protein 423; *SHISA9*, shisa family member 9; *TMEM132B*, transmembrane protein 132B; *GRID2*, glutamate ionotropic receptor delta‐type subunit 2; *STARD13*, StAR‐related lipid transfer domain containing 13; *DMD*, dystrophin; *DLC1*, DLC1 Rho GTPase activating protein; *LMCD1‐AS1*, LMCD1 antisense RNA 1; *RPS6KA2*, ribosomal protein S6 kinase A2; *SLC4A4*, solute carrier family 4 member 4; *ATP8A2*, ATPase phospholipid transporting 8A2.

## Supporting information

Supporting Information

Supporting Information

## Data Availability

Sequencing data could be downloaded from https://db.cngb.org/cnsa/gsa‐human (accession No.HRA003097). Relevant code was provided on GitHub (https://github.com/XiaoAILab/small‐eccDNA).
