# Supplementary material for: Small extrachromosomal circular DNAs as biomarkers for multi‐cancer diagnosis and monitoring
Source: Clin Transl Med. 2023 Aug 30;13(9):e1393. doi: 10.1002/ctm2.1393 (PMC10468585; doi:10.1002/ctm2.1393)
Supplement: Supplementary file 1 — Supporting Information [file CTM2-13-e1393-s001.docx]

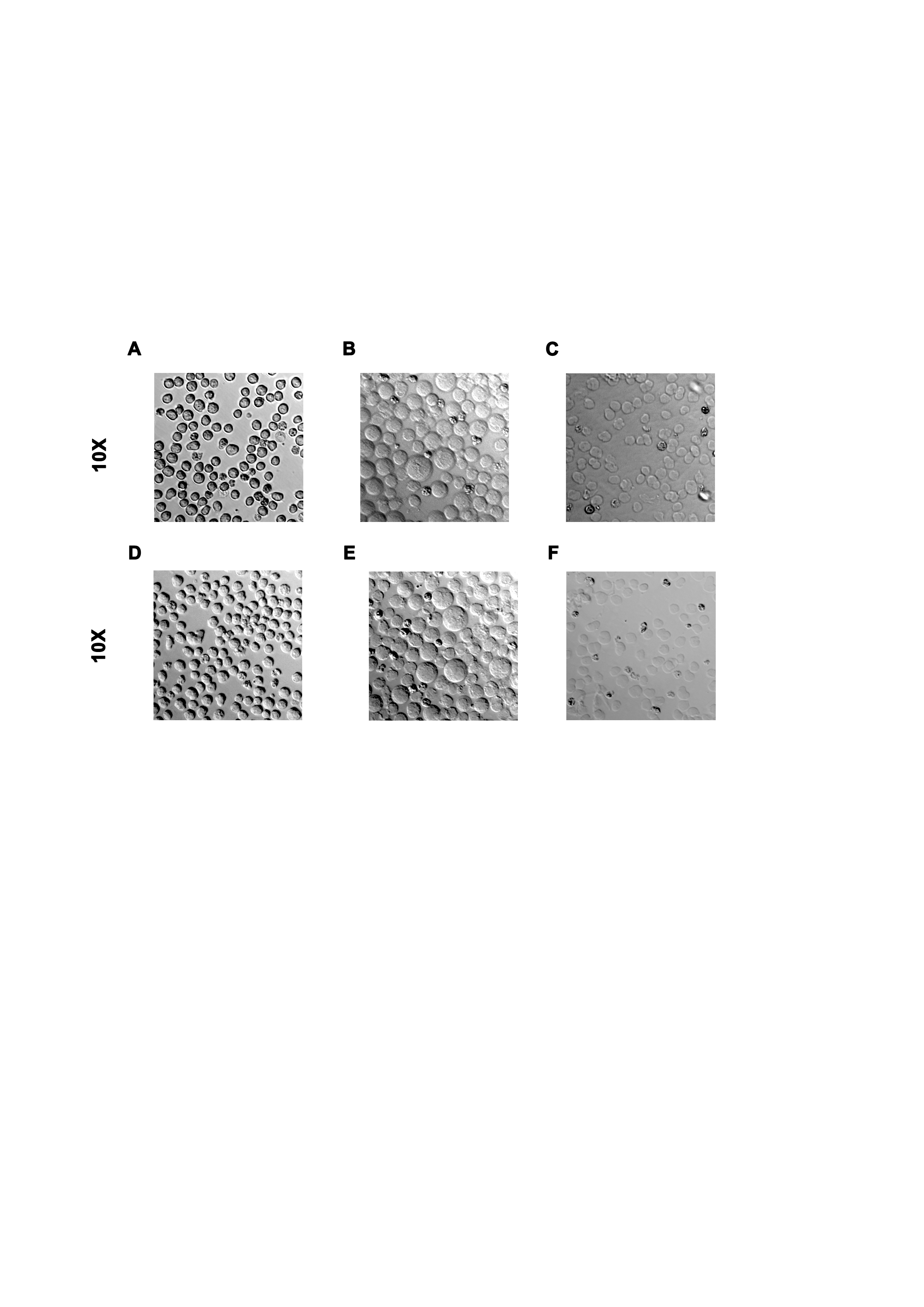


Supplementary Figure 1. Microscopic images (10X) of the cytoplasmic/nuclear separation step in our workflow, using HepG2 cells as an example. (A) Microscopic image of HepG2 cells in PBS buffer. (B) Microscopic image of HepG2 cells in a hypotonic buffer (containing 10 mM HEPES-KOH [pH 7.9], 1.5 mM MgCl_2_, 10 mM DTT, and 0.2 mM PMSF). (C) Microscopic image of HepG2 cells in the cytoplasmic membrane lysis buffer (containing 10 mM HEPES-KOH [pH 7.9], 1.5 mM MgCl_2_, 10 mM DTT, 0.2 mM PMSF, and 0.2% NP-40). (D) Microscopic image of single cells from liver tissues in PBS buffer. (E) Microscopic image of single cells isolated from liver tissues in the hypotonic buffer. (F) Microscopic image of single cells isolated from liver tissues in the cytoplasmic membrane lysis buffer.


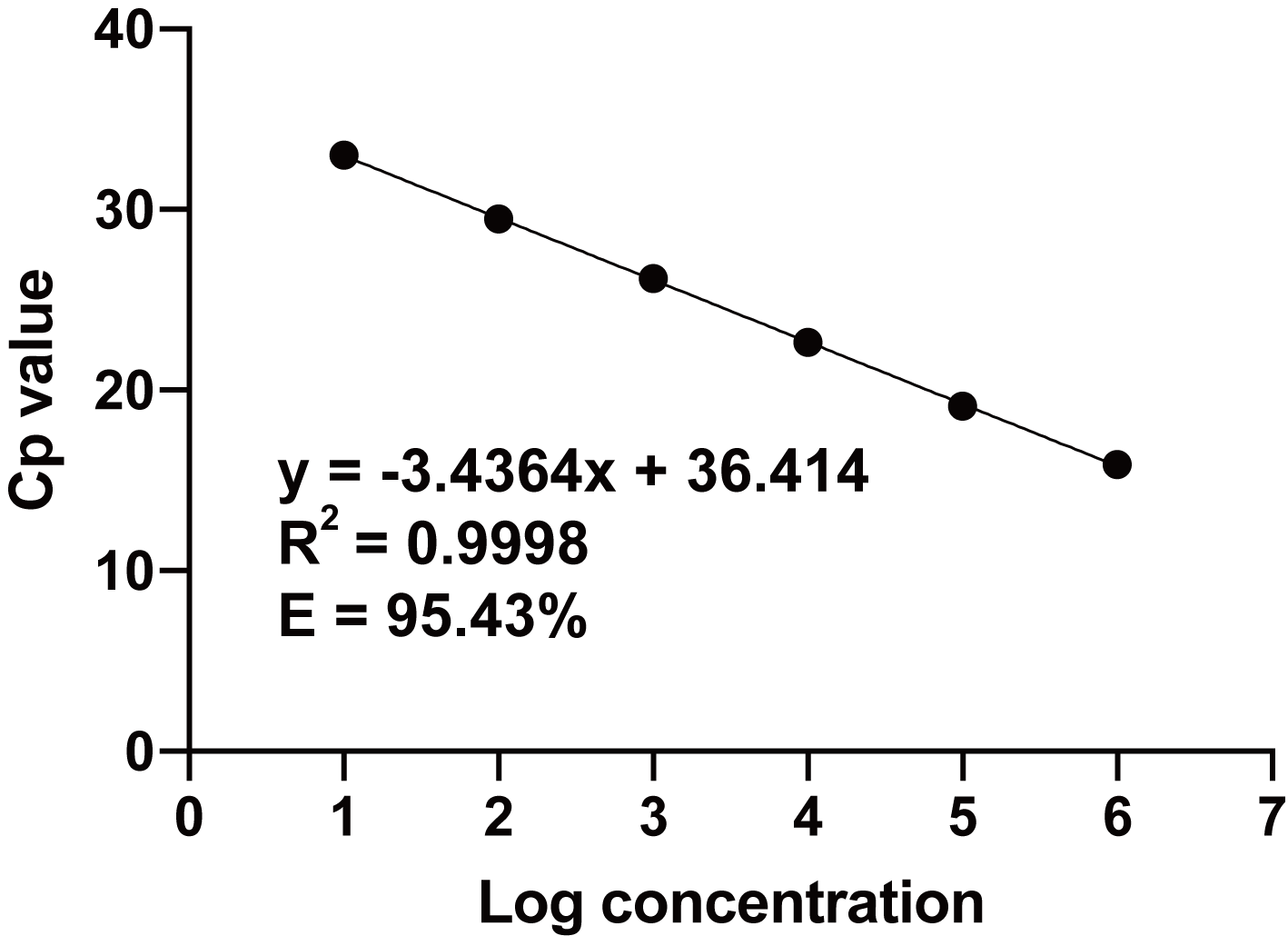


Supplementary Figure 2. Amplification efficiency Evaluation of primer for *COX5B* in qPCR.


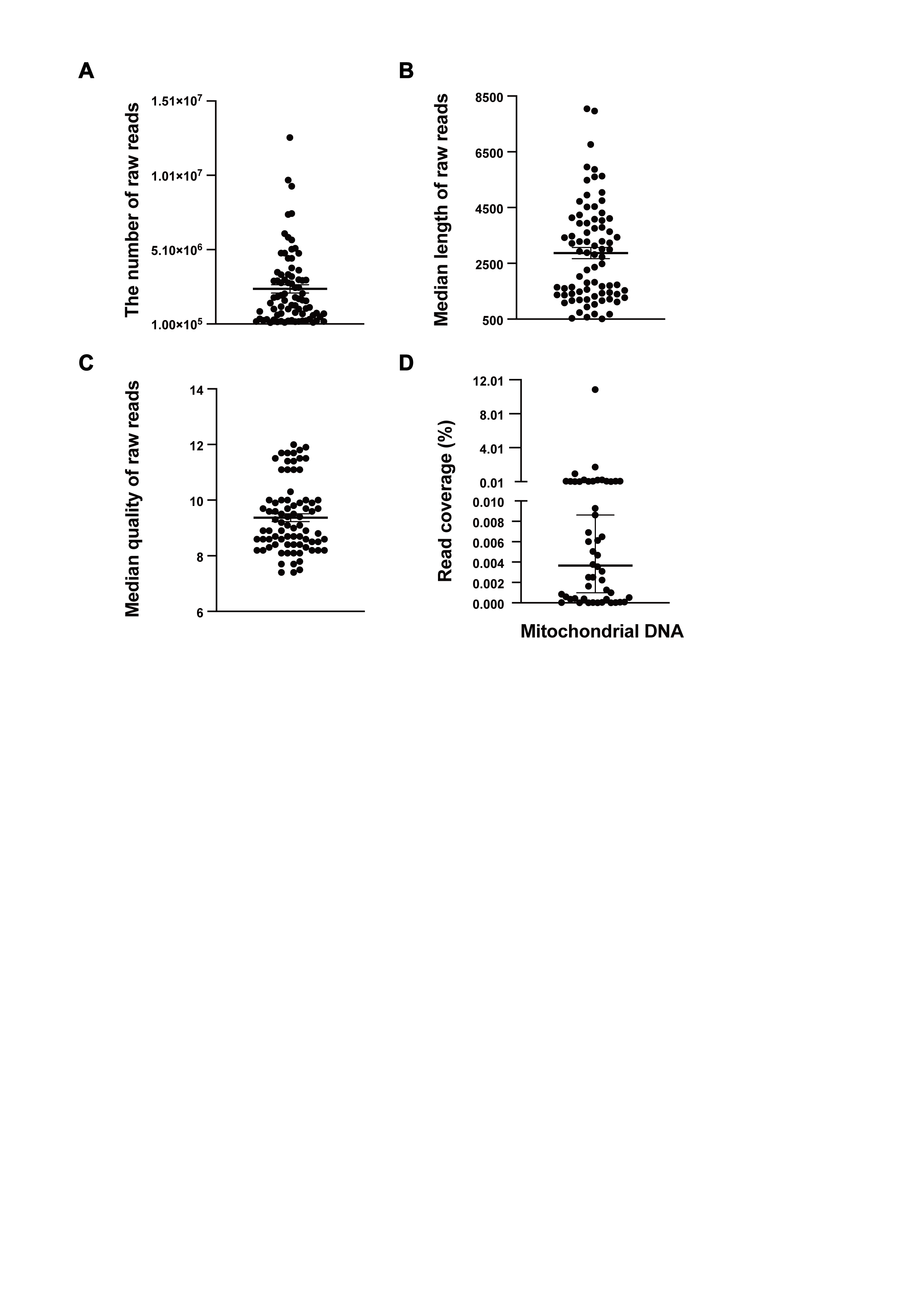


Supplementary Figure 3. Details for sequencing reads in each sample. (A) The number of raw reads. Data are expressed as mean$\pm$SEM. (B) The median length of raw reads. Data are expressed as mean$\pm$SEM. (C) The median quality of raw reads. Data are expressed as mean$\pm$SEM. (D) The percentage of mapped reads corresponding to mitochondrial DNA in raw reads. Data are expressed as median$\pm$95%Cl.


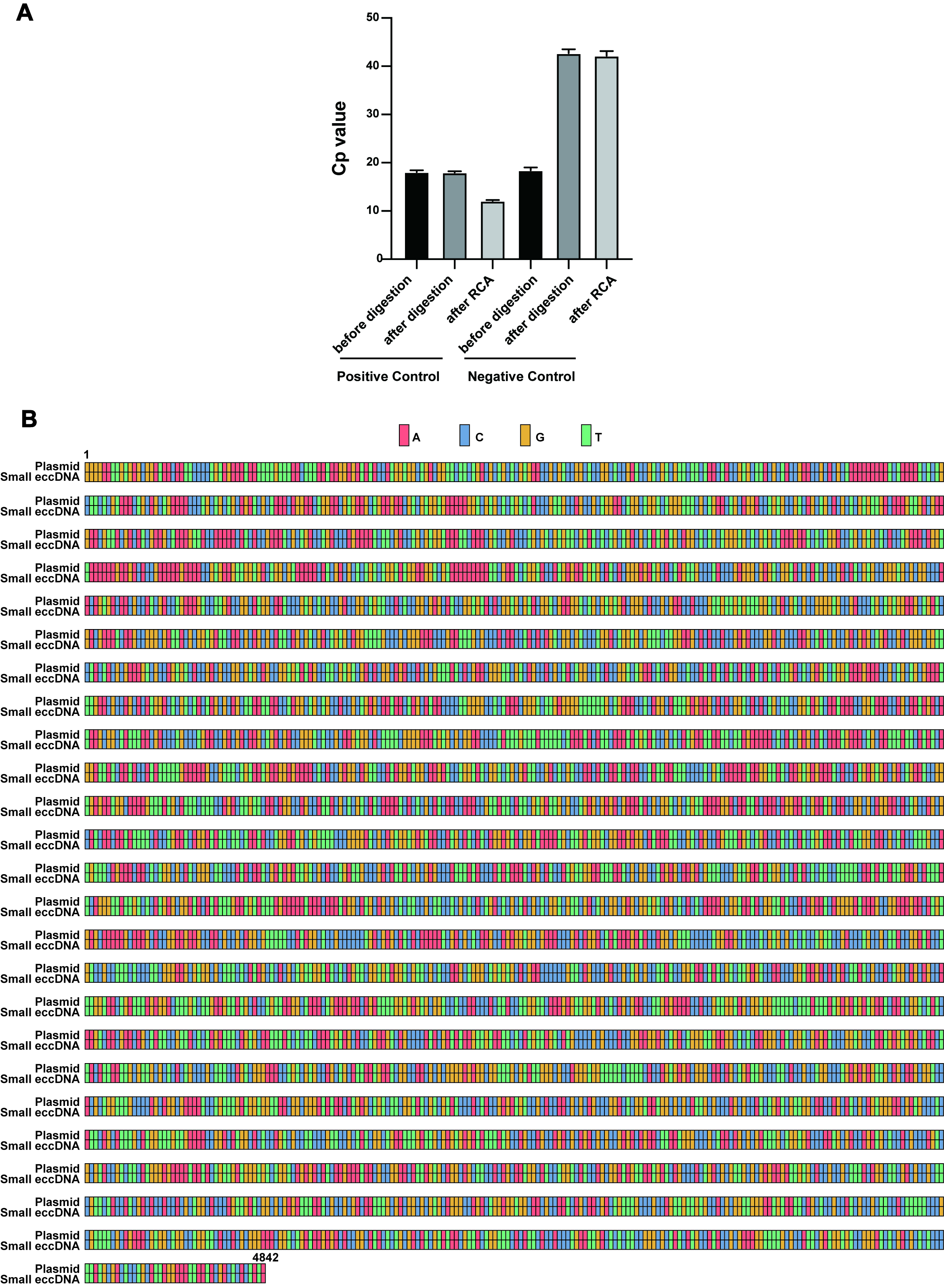


Supplementary Figure 4. A positive (plasmid) and negative control (linear PCR product) for the validation of our workflow starting from adding nuclear membrane lysis buffer. (A) the qPCR (5'-CAACCCGGTAAGACACGACT-3' and 5'-AAGGTTCGCTGAGCTACCAA-3') results before and after exonuclease digestion and after rolling circle amplification (RCA). Cp, crossing point. Data are expressed as mean $\pm$ SEM with three biological replicates. (B) The sequence alignment result between a small eccDNA identified in the positive control and the full-length sequence of the plasmid.


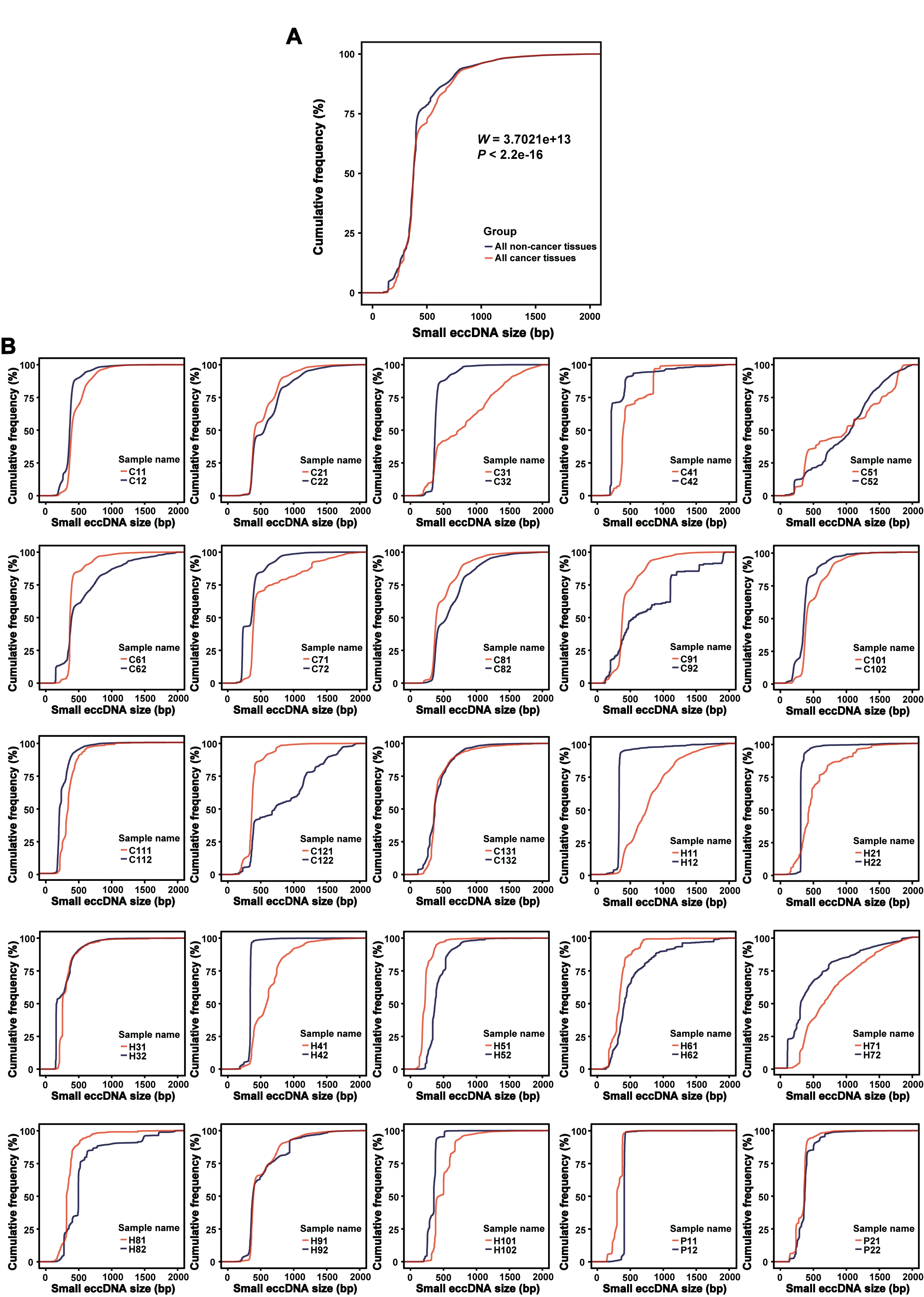


Supplementary Figure 5. (A) Cumulative frequency plots of small eccDNAs (shorter than 2000 bp) detected in non-cancer and cancer tissues. (B) Cumulative frequency plots of small eccDNAs (shorter than 2000 bp) detected in each tissue sample.


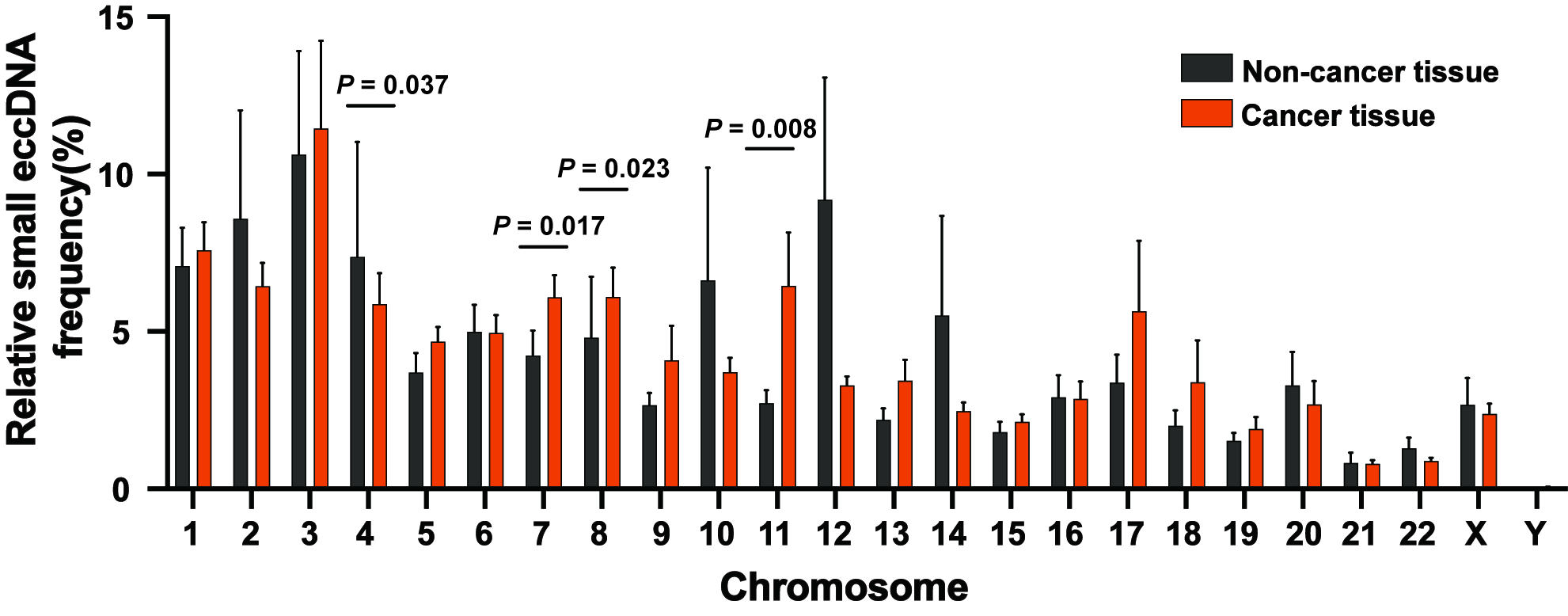


Supplementary Figure 6. Distribution of small eccDNAs in non-cancer and cancer tissues on each chromosome. Differences between groups were compared by Wilcoxon signed-rank test.


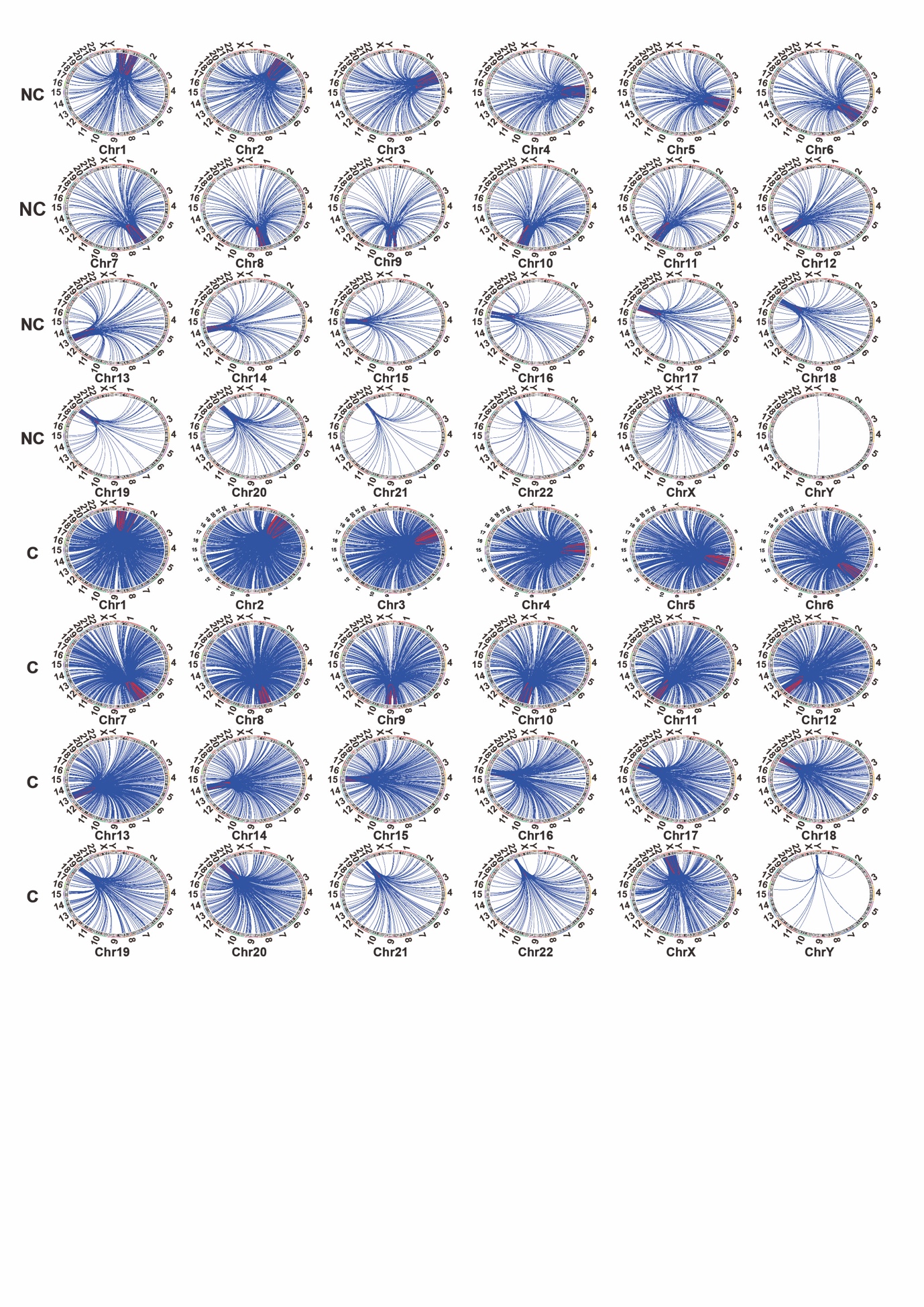


Supplementary Figure 7. Circle plot showing the chromosomal origin of all two-fragment small eccDNAs (2f small eccDNA) in non-cancer (NC) and cancer (C) tissues, respectively. Chr, chromosome. One line represents a type of 2f small eccDNA events.


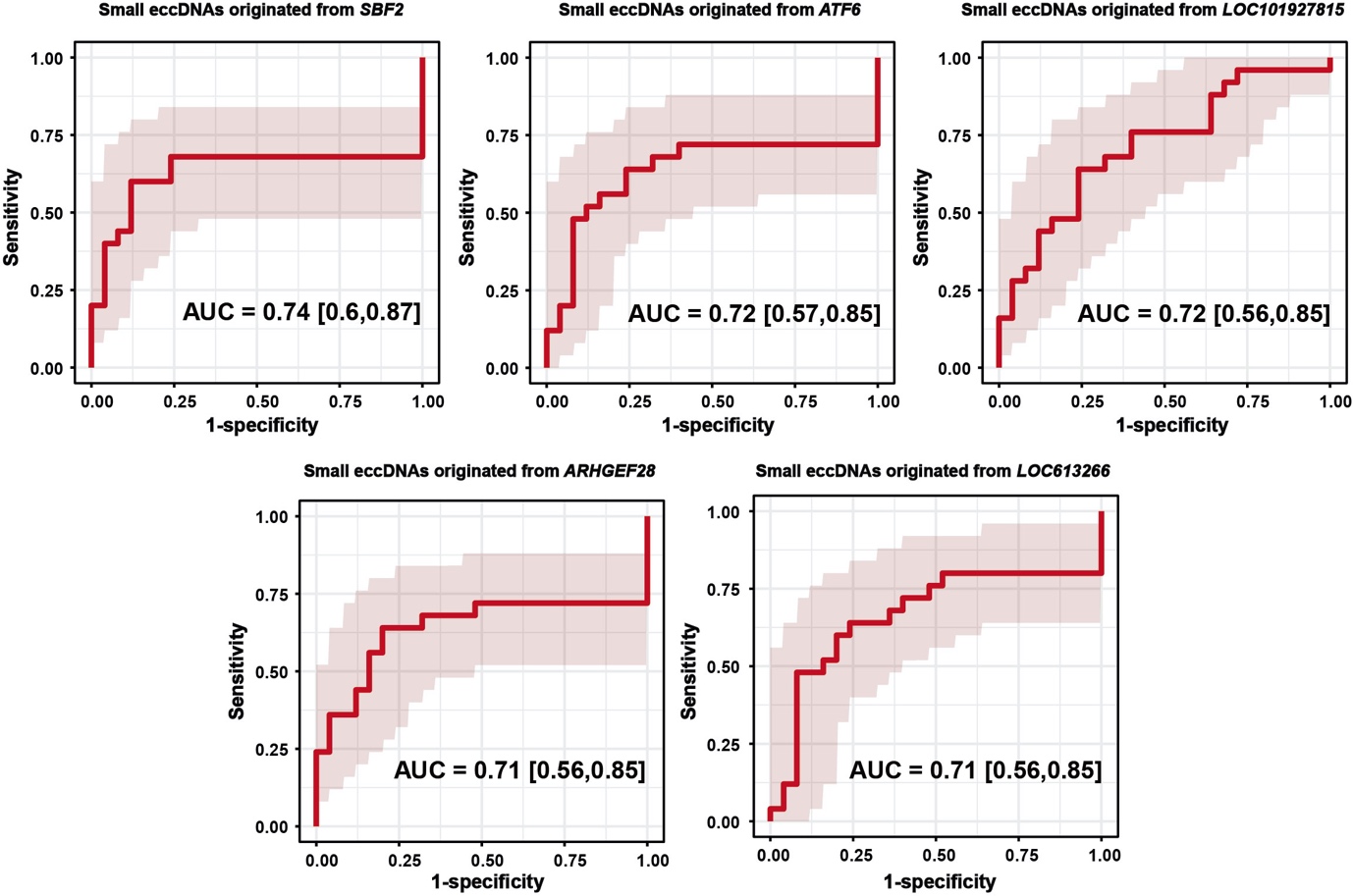


Supplementary Figure 8. The multi-cancer diagnostic value in tissues of the proportion of small eccDNAs originated from some genes (all AUC > 0.7 and *P* < 0.05). *SBF2*, SET binding factor 2; *ARHGEF28*, Rho guanine nucleotide exchange factor 28; *ATF6*, activating transcription factor 6; AUC, the area under the ROC curve.


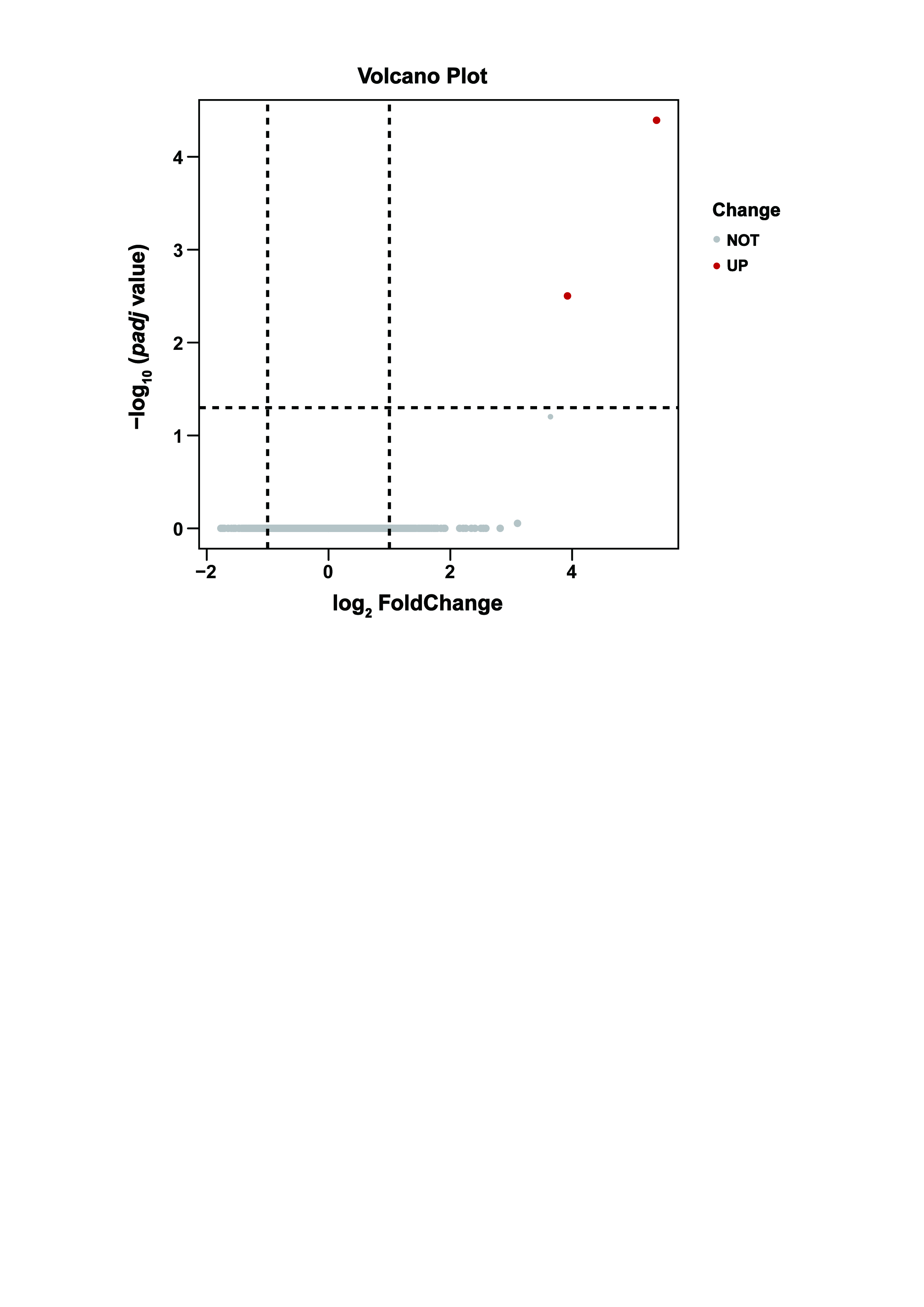


Supplementary Figure 9. Volcano plot of the difference in the proportion of small eccDNAs originated from specific genes between cancer plasma and cancer tissues. Grey dots represent low expressed genes in cancer tissues.


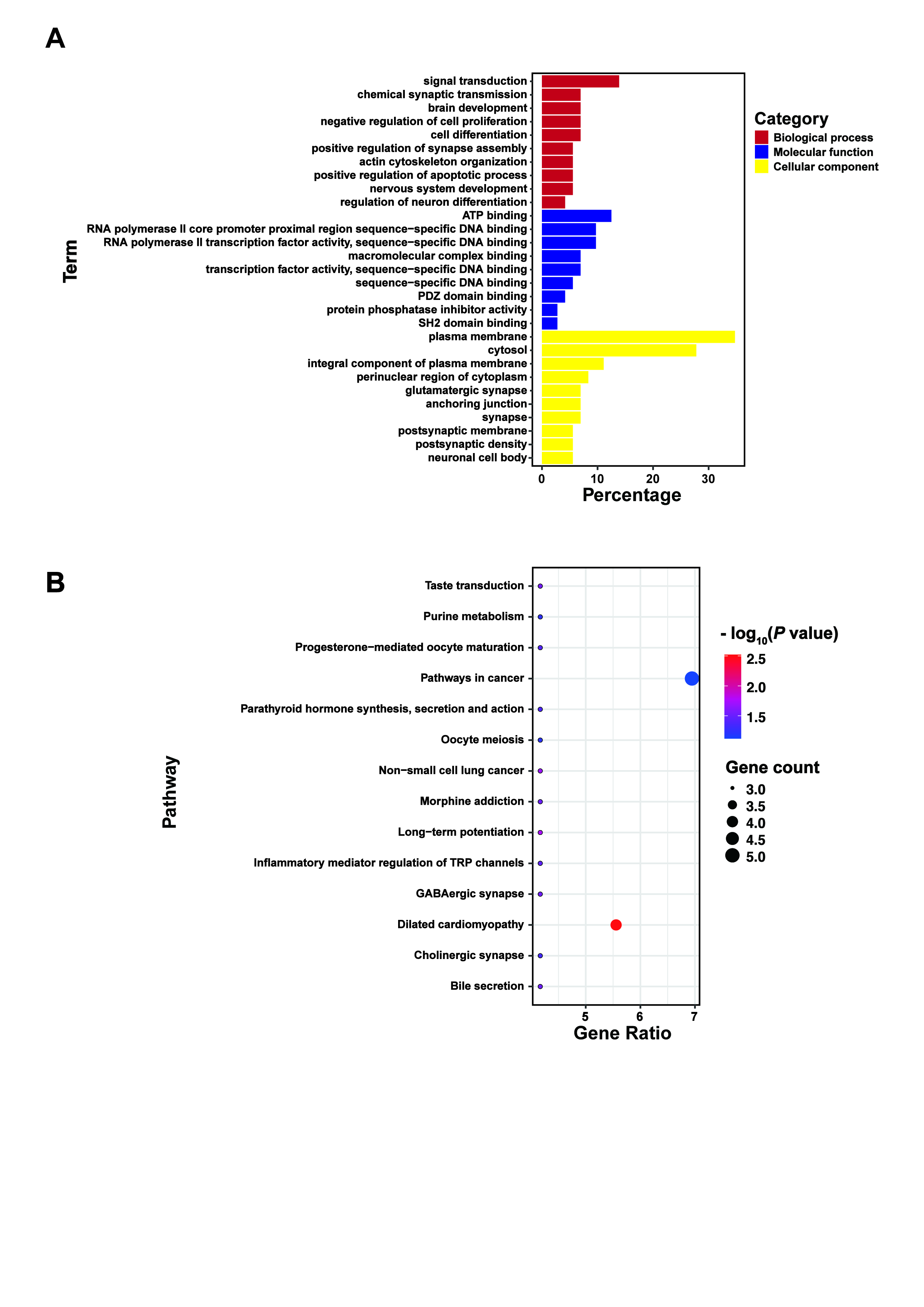


Supplementary Figure 10. Functional annotation of shared small eccDNA-associated genes between cancer plasma and cancer tissues. (A) Gene Ontology (GO) analyses of shared small eccDNA-associated genes between cancer tissues and cancer plasma using DAVID. (B) Kyoto Encylopedia of Genes and Genomes (KEGG) analyses of shared small eccDNA-associated genes between cancer tissues and cancer plasma using DAVID.


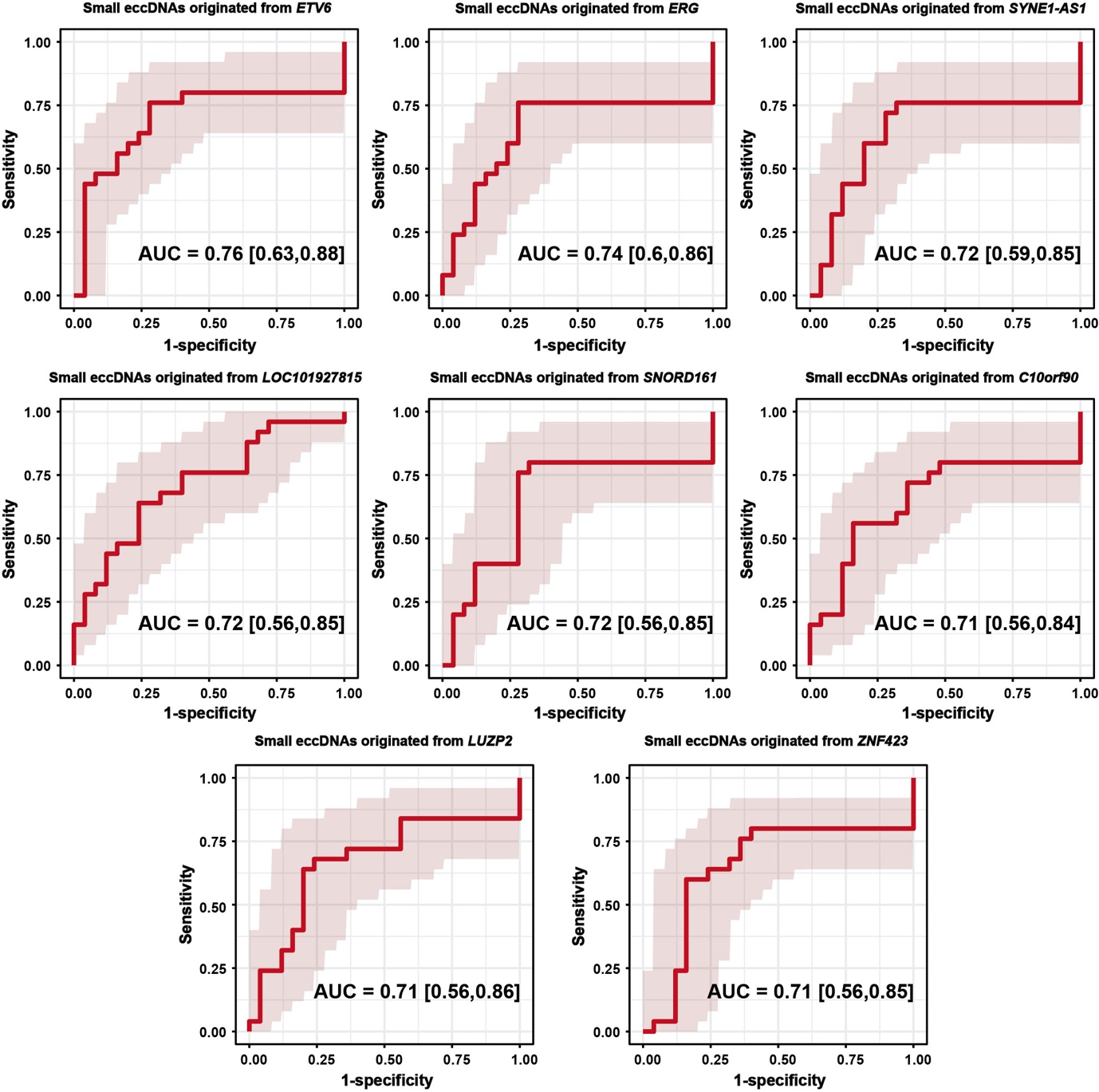


Supplementary Figure 11. The multi-cancer diagnostic value in tissues of the proportion of small eccDNAs originated from some genes (all AUC > 0.7 and *P* < 0.05). *ETV6*, ETS variant transcription factor 6; *ERG*, ETS transcription factor ERG; *SYNE1-AS1*, SYNE1 antisense RNA 1; *SNORD161*, small nucleolar RNA, C/D box 161; *C10orf90*, chromosome 10 open reading frame 90; *LUZP2*, leucine zipper protein 2; *ZNF423*, zinc finger protein 423; AUC, the area under the ROC curve.
