# Supplementary material for: Small extrachromosomal circular DNAs as biomarkers for multi‐cancer diagnosis and monitoring
Source: Clin Transl Med. 2023 Aug 30;13(9):e1393. doi: 10.1002/ctm2.1393 (PMC10468585; doi:10.1002/ctm2.1393)
Supplement: Supplementary file 2 — Supporting Information [file CTM2-13-e1393-s002.docx]

Supplementary Table 1. Clinical information on tissue samples and plasma samples.


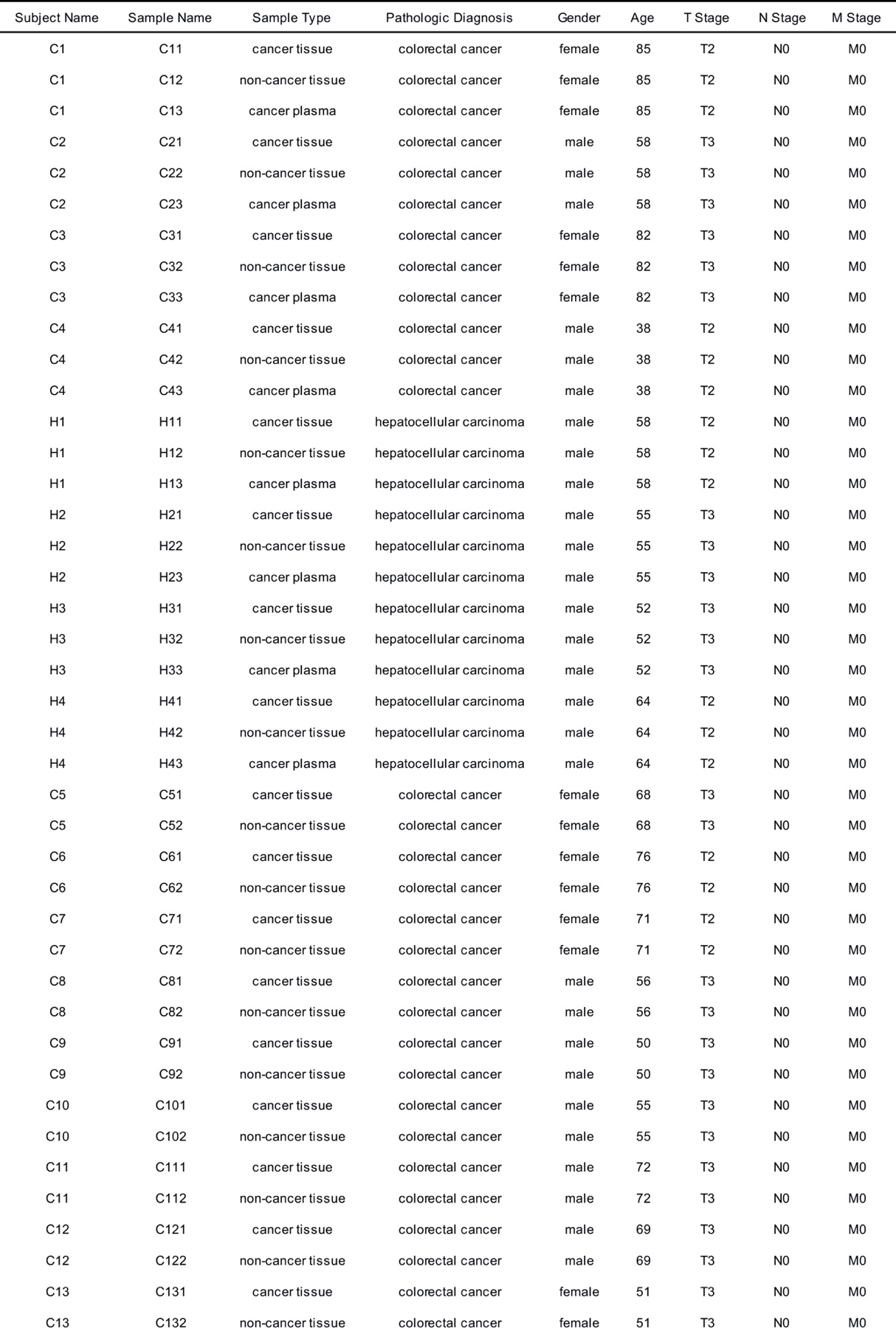

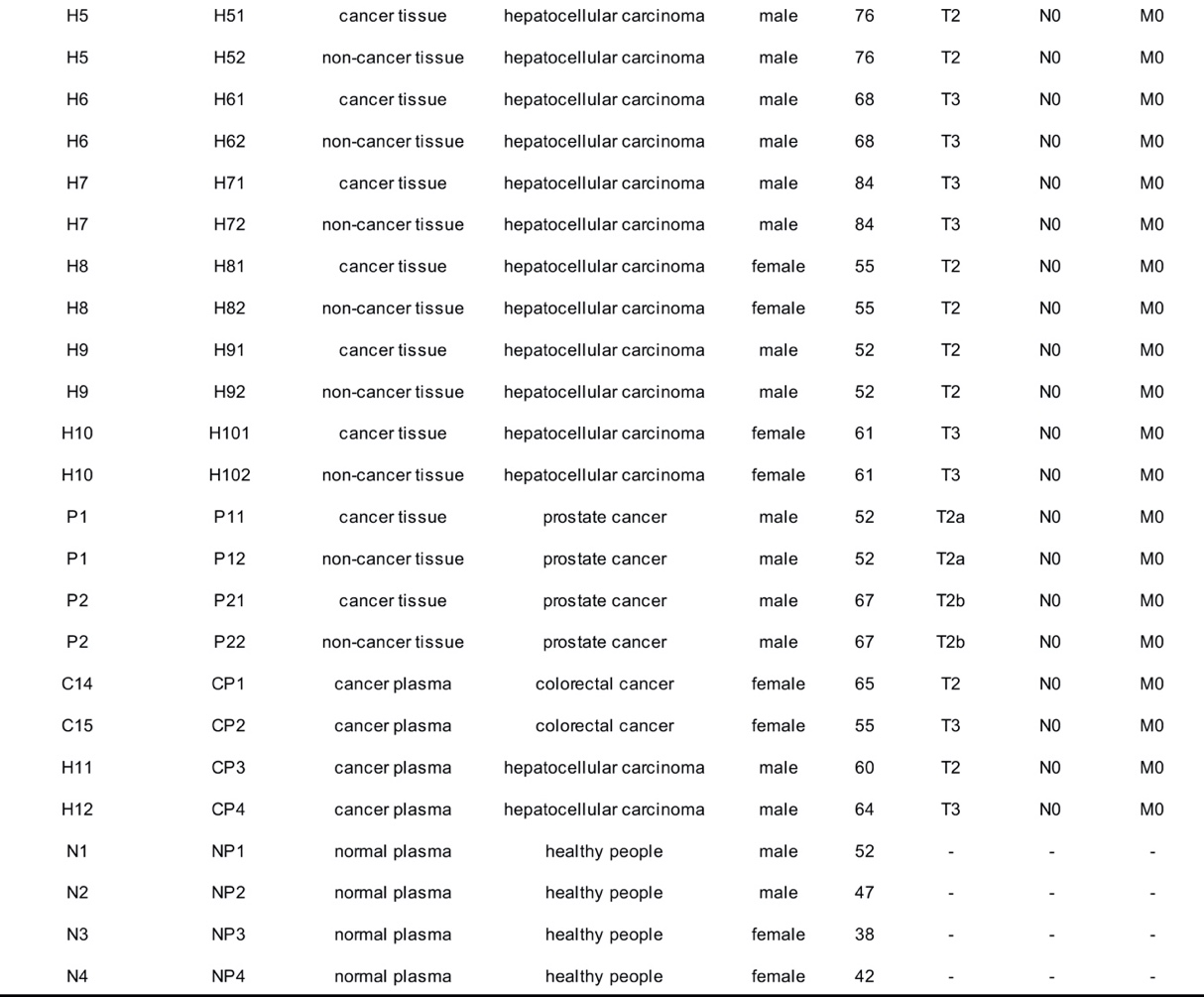


Supplementary Table 2. Clinical information on an independent validation cohort.


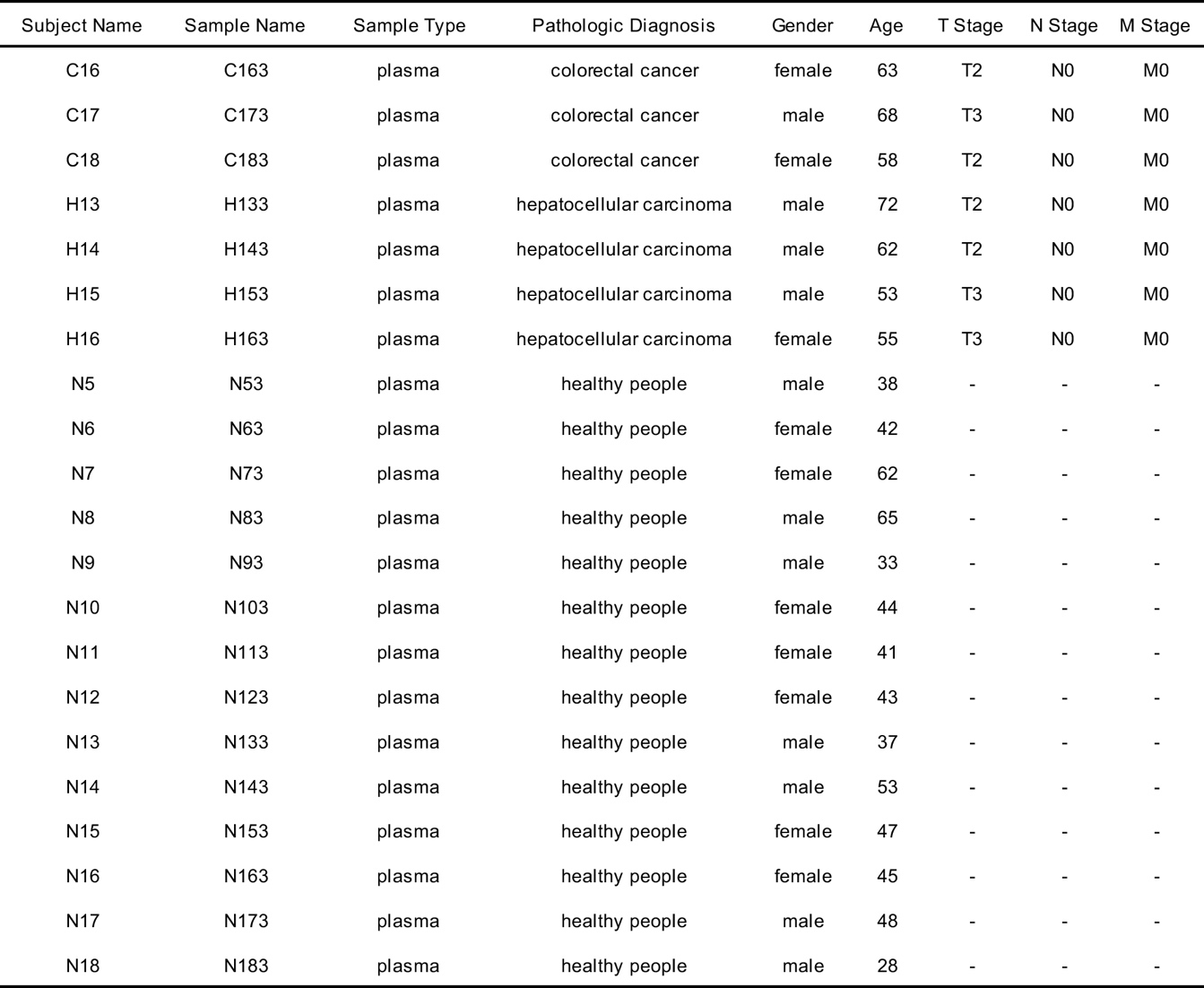


Supplementary Table 3. Quantitative PCR (qPCR) assessment of the crossing point (Cp) value of *COX5B* before and after treatment with exonuclease III and lambda exonuclease. The limit of detection for Cp value was 45.


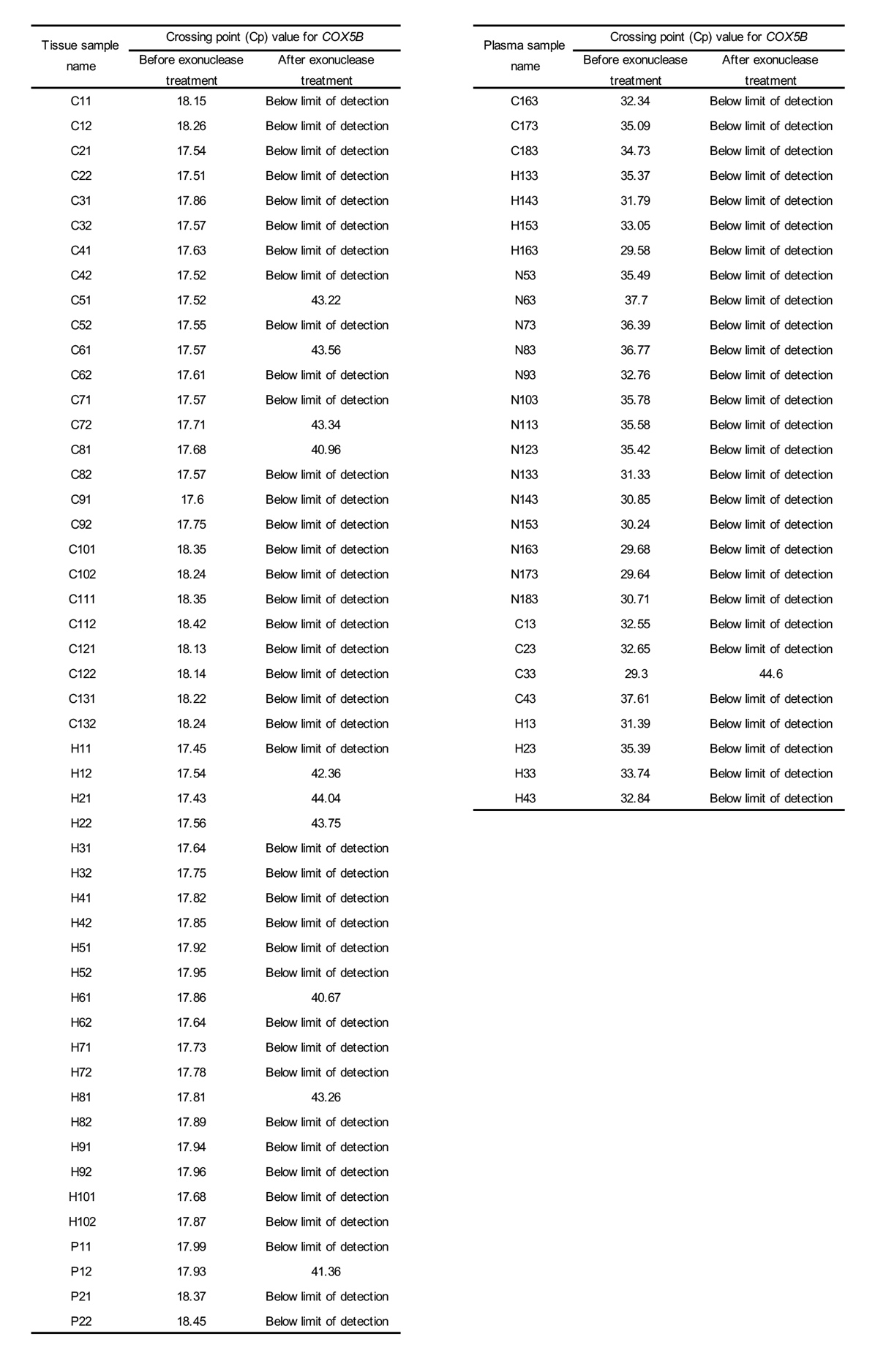


Supplementary Table 4: Sanger sequencing results of junction sites and lists of primers were used to validate small eccDNAs in tissues that was randomly selected from the sequencing results of the novel sequencing workflow. The red arrow points to the junction sites of small eccDNAs. Sample name represents the name of the sample used for small eccDNA validation.


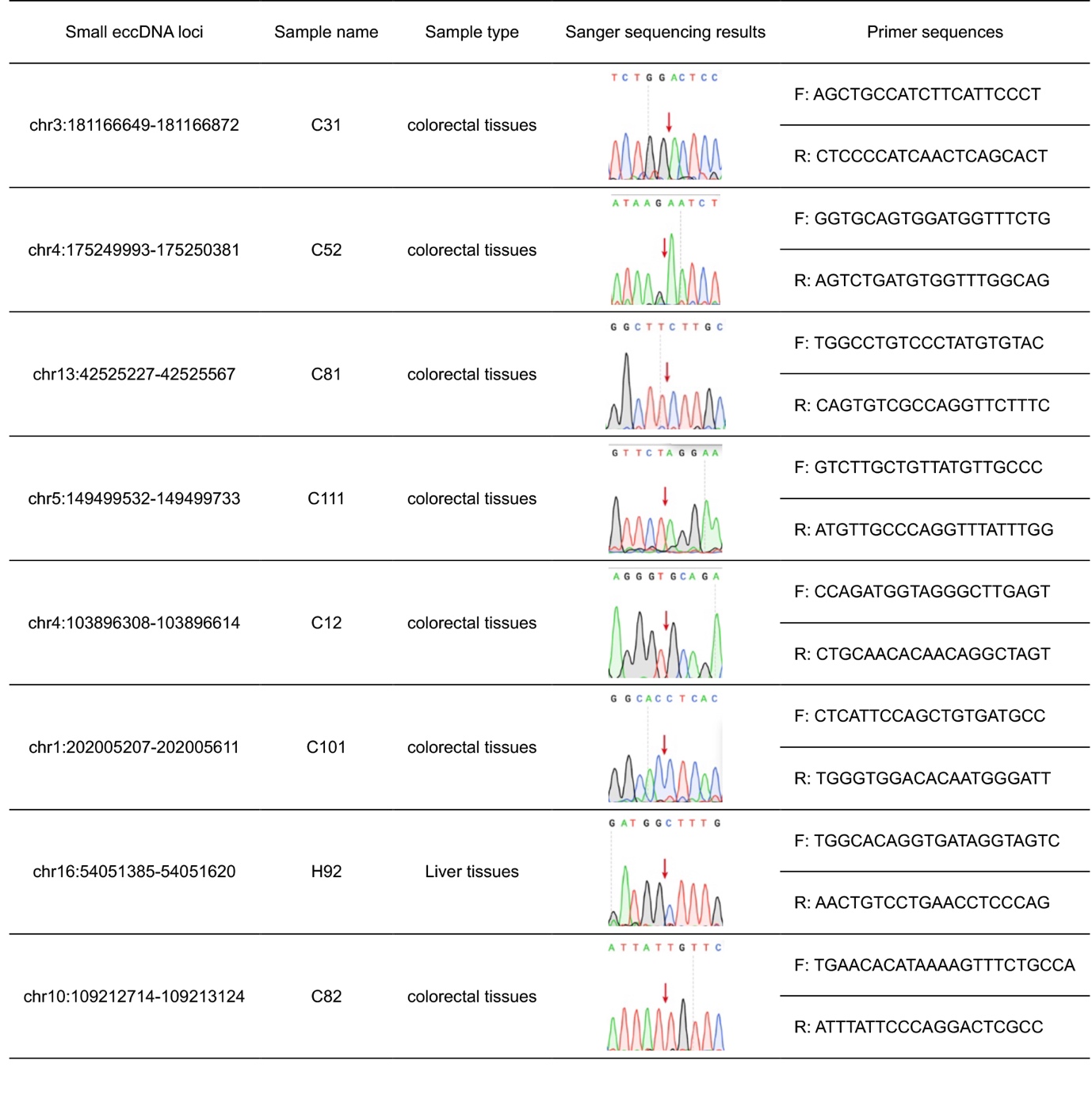


Supplementary Table 5: Sanger sequencing results of junction sites and lists of primers were used to explore whether the shared small eccDNA detected by high-throughput sequencing in eight cancer plasma samples was present in other cancer plasma and normal plasma. The red arrow points to the junction sites of small eccDNAs.


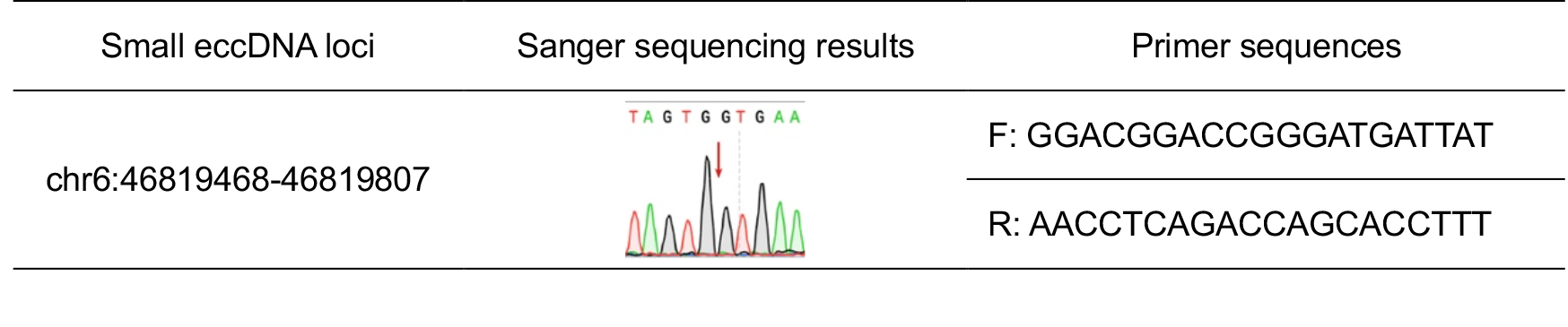


Supplementary Table 6: Sanger sequencing results of junction sites and lists of primers were used to validate three shared small eccDNAs between cancer tissue and cancer plasma, which was not found in non-cancer tissue. The red arrow points to the junction sites of small eccDNAs.

**
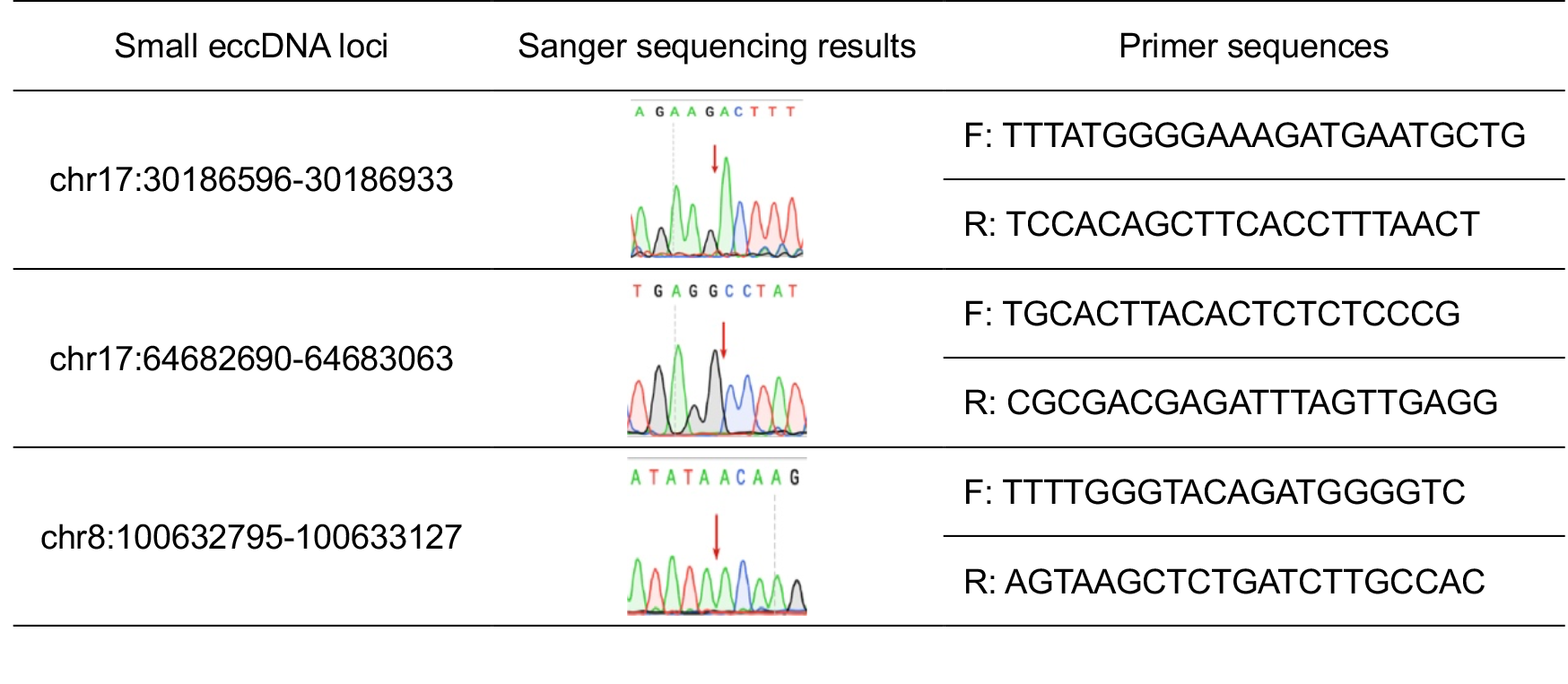
**

Supplementary Table 7. The percentage of small eccDNA biomarkers detected in cancer (C) and non-cancer (NC) tissues and their paired CEA/CA19-9 levels.


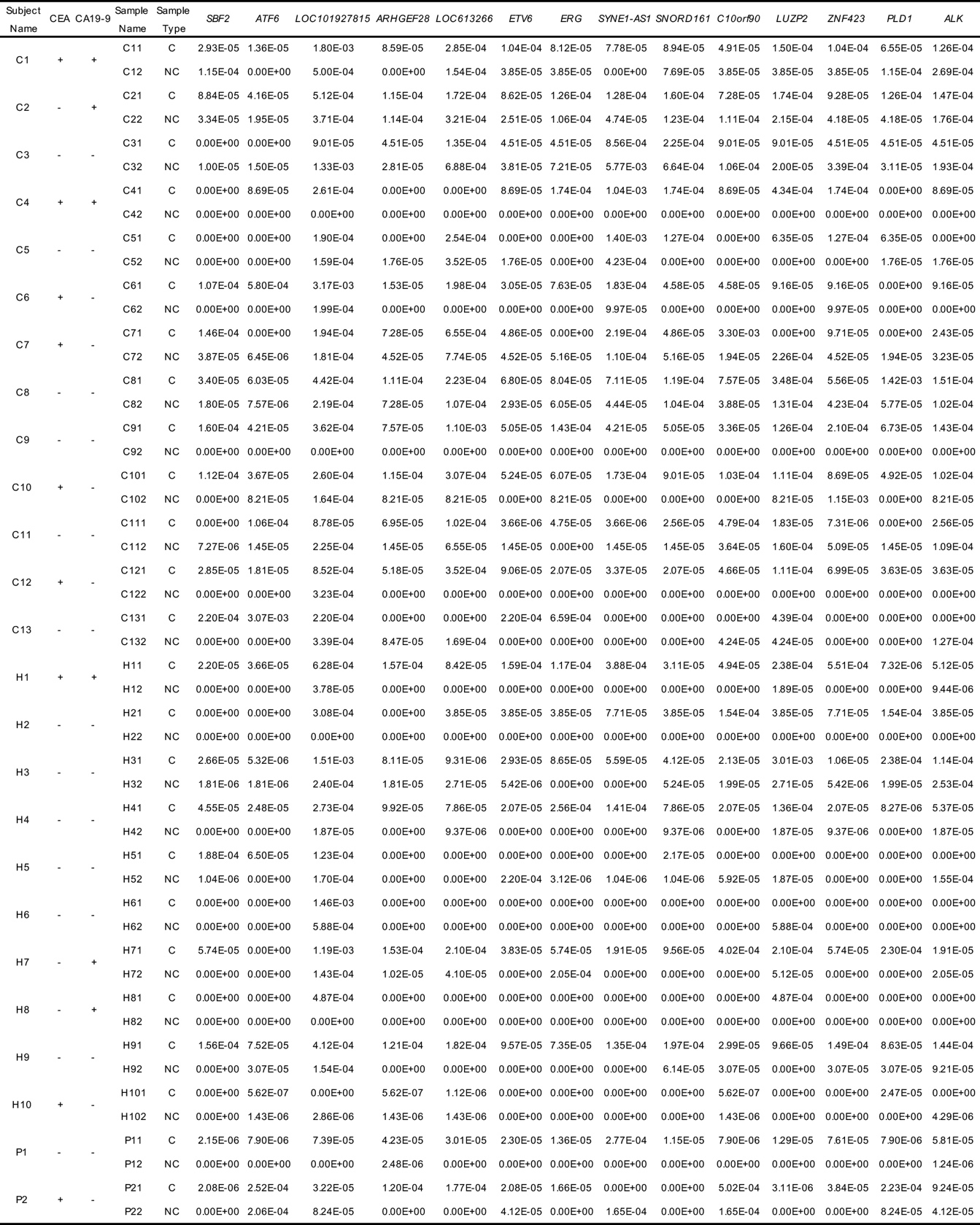


Note: The cut-off point for CEA and CA19-9 was 5 μg/L and 37 kU/L, respectively. +, positive; -, negative.

Supplementary Table 8. The percentage of small eccDNA biomarkers detected in plasma samples and their paired CEA/CA19-9 levels.


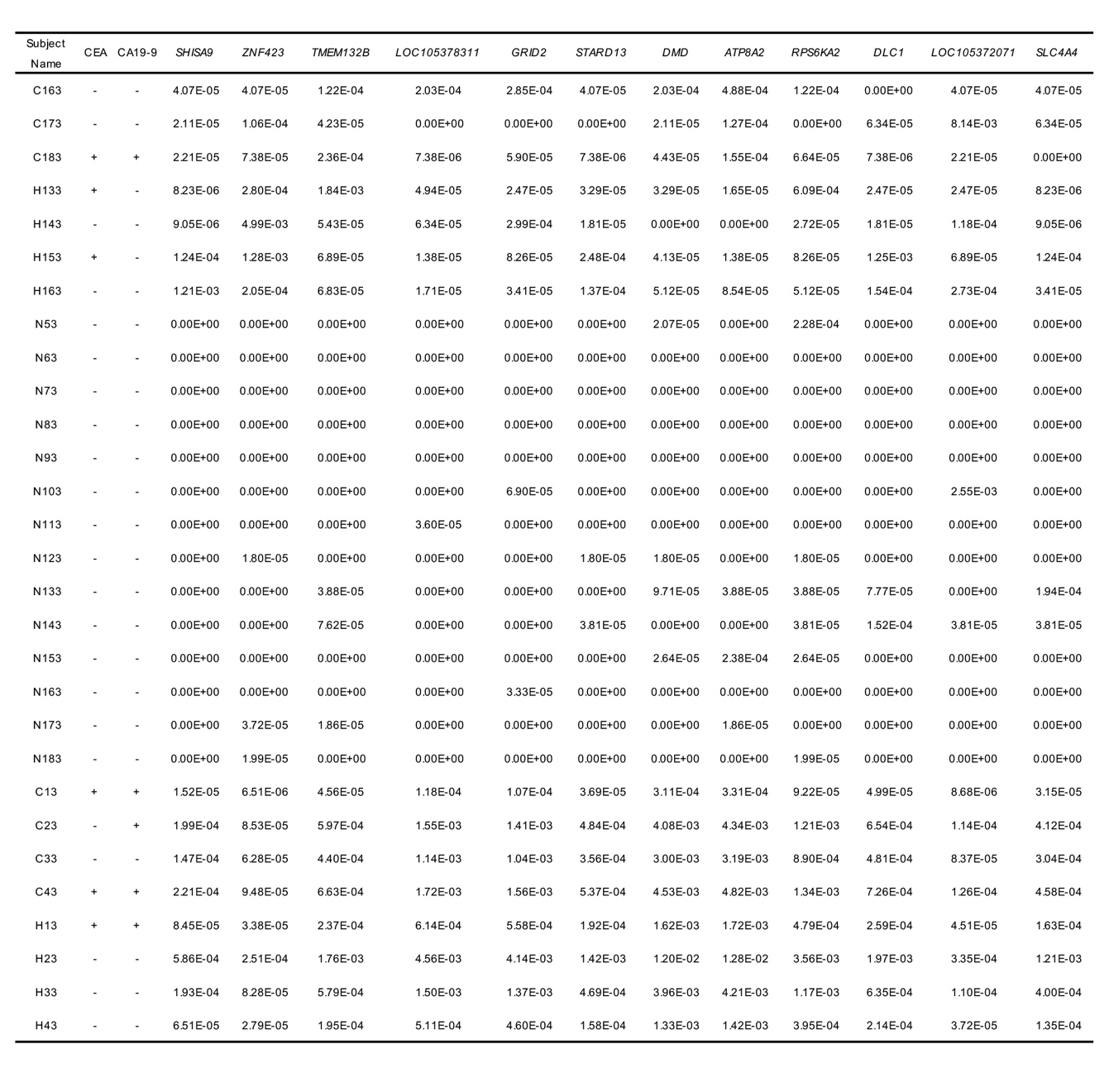
Note: The cut-off point for CEA and CA19-9 was 5 μg/L and 37 kU/L, respectively. +, positive; -, negative.
